# Modeling the global potential of smart farming innovations

**DOI:** 10.1073/pnas.2530081123

**Published:** 2026-10-19

**Authors:** Shahrear Roman, Hadi Hadi, Wyclife Agumba Oluoch, David Zilberman, Robert Finger, David Wuepper

**Affiliations:** ^a^ Land Economics Group, Institute for Food and Resource Economics, University of Bonn, Bonn 53115, Germany; ^b^Department of Agricultural and Resource Economics, University of California Berkeley, Berkeley, CA 94720; ^c^ Agricultural Economics and Policy Group, Department of Management, Technology, and Economics, ETH Zürich, Zürich 33 8092, Switzerland

**Keywords:** smart farming innovations, digital technology, sustainability challenges, technology fit

## Abstract

Smart farming innovations can improve agricultural productivity, resilience, and environmental sustainability, yet their real-world impact hinges on their diffusion dynamics, which in turn depend on their economic viability. We present here a global assessment of the profitability of smart farming innovation across croplands at a spatial resolution of ~5 km from the farmers’ and the societal perspectives. We cover application mapping for precision agriculture, automated application of inputs, autonomous weeding machines, GPS steering assistance systems, and autonomous irrigation systems. For this, we compiled a globally comprehensive dataset of smart farming innovation adoption locations (N = 4,422) based on a combination of a survey (N = 2,677) and a meta-analysis (N = 1,745) and combined them with remotely sensed and statistical data to train an ensemble of five geospatial machine learning models (generalized linear models, random forests, support vector machines, maximum entropy, and boosted regression trees), which show the global distribution of the different innovations’ profitability and its overlap with major environmental issues the innovations can help to mitigate. Simulations of future socioeconomic pathways combined with assumptions about future agri-environmental policies reveal that, especially in middle-income countries, the potential of smart farming will grow over time.

## Significance

This study presents an assessment of the potential of smart farming innovations, based on an ensemble of machine learning algorithms trained on 4,422 observations from a global survey and meta-analysis. We find policy- and business-relevant patterns and show regions in which the profitability of smart farming innovation overlaps with environmental issues they can help to address. Moreover, our future projections under the Shared Socioeconomic Pathway-2 and contrasting environmental governance scenarios indicate that smart farming potential will increase globally, though with considerable heterogeneity: Gains are most pronounced in South America, Eastern Europe, and South and East Asia, while sub-Saharan Africa shows persistently lower potential. Proactive environmental policies could accelerate smart farming diffusion in regions where sustainability cobenefits are greatest.

Agriculture is confronted with a series of interrelated challenges, including a rapidly growing demand for food—e.g., driven by rising incomes (1) and population growth (2), reduced production potential, e.g., due to climate change and land degradation (3–5), and economic challenges, such as shortage of agricultural labor (6, 7). Meanwhile, demographic stagnation and aging populations in many high-income countries further compound agricultural labor constraints while creating regionally divergent demand trajectories (8). The global diffusion of the intensive use of agrochemicals has led to a notable enhancement that has greatly improved global agricultural productivity (9–11). However, it has also led to substantial ecological and health costs (12, 13). Agriculture must urgently become more sustainable, as it is currently among the main drivers of the global decline in biodiversity and ecosystem services (14, 15). The challenge is that this increase in environmental sustainability must be coupled with an increase in food production. Farmers need to produce more with less (16), and this must happen quickly (17, 18). Considerable hope rests on the development and diffusion of smart farming innovations, which aim to make agricultural practices more targeted, flexible, and locally adapted, overall allowing much higher levels of efficiency (16, 19, 20). These technologies can even be used beyond production, for example for “precision conservation” (21). Ultimately, however, the potential of smart farming innovations is shaped by their economic viability—if they are not profitable, they are unlikely to diffuse widely and have large global impacts (22). Thus, we undertook an extensive global survey from November 2023 to August 2024 to obtain geolocations where smart farming systems are currently profitable. We employed a purposive sampling strategy (23, 24) to identify key informants with specialized knowledge of smart farming technologies across three informant groups: Agricultural technology providers, farmers implementing these technologies, and researchers. To expand geographic and technological coverage, we utilized snowball sampling (25), whereby initial respondents from all three informant groups were requested to recommend other qualified experts within their professional networks. Then, we combined this large-scale survey with a comprehensive meta-analysis on agricultural technology adoption studies (26) to train geospatial machine learning (ML) techniques to predict the profitability of various smart farming innovations focusing on crops, to see where in the world they hold potential, where this overlaps with environmental issues they could help to address, and to see how much continued socioeconomic development (27) coupled with trends in agri-environmental policies (28) is projected to reshape these patterns in the future.

Smart farming innovations (SFI) are a range of advanced precision agriculture technologies and practices—which are often bundled—designed to enhance farming efficiency, productivity, and environmental sustainability (29). Through experts’ interviews from academia, business, and practice, we first developed a long list of possible smart farming innovations that could be included in this study. We then iteratively narrowed the list down to five key technologies that represent the range of technologies and applications. All of these technologies are based on automation, sensing, and increased precision and have the potential to improve the efficiency of the use of agrochemicals, water, and other inputs. Finally, in this study, we consider the following smart farming innovations that have a potential relevance for agriculture globally, can help to address agri-environmental problems and are readily available in the market: i) application mapping for precision agriculture, ii) automated application of variable inputs (VRT), iii) autonomous weeding machines, iv) GPS steering assistance systems, and v) autonomous irrigation systems. Notably, the terms “automated” and “autonomous” as used herein encompass the full spectrum of automation, recognizing that current agricultural technologies typically involve varying degrees of human oversight and decision-making. These innovations encompass various dimensions of digitalization in agriculture, including georeferencing and location technologies such as GPS steering systems, digital diagnostic tools such as sensors, scanners on machinery, and remote sensing via drones and satellites for information gathering, and applicative tools that enable site-specific management actions like variable rate application of fertilizers and pesticides (16, 29). Additionally, smart farming incorporates automated and autonomous processes, including robotic systems for tasks such as weeding and seeding, as well as networks, information, and communication technologies that interconnect sensors, machines, operations, and people (16). By integrating real-time sensor data to prevent water waste and lowering operational costs in regions facing economic water scarcity, smart irrigation systems can enable sustainable agricultural intensification where conventional efficiency measures have historically struggled (30, 31). These technologies span a wide spectrum of development and diffusion stages—from solutions like GPS and VRT that are in some regions relatively well established already, to emerging innovations that are not yet widely used, up to such innovations as autonomous weeding robots, which have just started to be adopted (16, 32–36). A common observation is that diffusion patterns are correlated with income patterns, i.e., we observe more diffusion in higher income countries, and less diffusion in lower income countries (37).

A unifying element of the different smart farming innovations is the reduction of input waste (16, 36, 38). For example, variable rate technology allows for the delivery of exactly tailored amounts of water, nutrients, pesticides, or fertilizer, based on spatial and temporal crop needs, thereby reducing the inefficiencies of uniform field application, which regularly leads to over- and underapplication of inputs (20, 27, 28). Similarly, GPS steering assistance systems help to avoid wasting fuel and agricultural inputs by making the driving more precise and avoiding overlaps (39); and weeding robots can now detect and eliminate weeds through targeted herbicide application, or even without herbicides, e.g., using mechanical weed control or lasers, reducing reliance on indiscriminate spraying of entire fields (40). Likewise, autonomous irrigation systems allow more efficient water management, providing water to the plants when and where it is needed, again allowing to better match plant needs and in this case, save water (41). Collectively, these input efficiency features of smart farming innovations align with the principles of climate-smart agriculture by simultaneously enhancing productivity, reducing greenhouse gas emissions through lower fuel consumption and lower input use, decreased nitrogen losses, and reduced indirect energy footprints from agrochemical production, and building resilience to resource constraints (42–44). For example, GPS guidance systems reduce fuel use by 6 to 25% (45, 46), while variable rate nitrogen application has demonstrated N_2_O emission reductions of up to 34% (47, 48). Moreover, some smart farming innovations may reduce labor demand and thus address future agricultural labor constraints (29, 49).

While these smart farming innovations offer important private and public benefits, there are important constraints to their widespread adoption, including the fact that farmers need to pay for them, but a share of the environmental and health benefits is reaped by society (22). Frequently, their adoption is hampered by a lack of economic viability (50, 51). These economic hurdles for farmers are often also interlinked with infrastructural and regulatory hurdles, as well as a lack of appropriate extension and financial services. Often, larger farms are more likely to embrace new technologies (52), as substantial capital investment costs for equipment, along with access to information such as georeferencing services, can pose barriers (53). Additionally, the complexities of smart farming production systems entail learning costs (54), while uncertainties regarding the potential benefits may diminish adoption incentives (55, 56). Finally, the rapid pace of innovation introduces technology risks that can deter adoption (37). So far, in the United States, United Kingdom, Australia, and Denmark, the adoption of VRT remains heterogeneous among the countries and crops; for example, only cereal and oilseed farms in the Eastern United Kingdom exceed a 42% adoption rate, while in the USA, VRT adoption has plateaued at 31 to 37% of planted area for corn, soybeans, and winter wheat (57, 58). Similarly, in Australia, VRT fertilizer was used by only 11% of large grain farms nationally in 2021, while Denmark shows a notable disparity where VRT adoption appears low by farm count (13%) but covers 37% of crop area in 2023, reflecting higher adoption among larger operations (58).

VRT uptake also changes over time, for example, in the USA, the adoption of VRT increased significantly between 1998 and 2023: Corn/maize rose from 8 to 37%, soybeans from 7 to 34%, peanuts from 1 to 21%, and winter wheat from 2 to 21% (58). In the United Kingdom, VRT increased from 13 to 25% between 2009 and 2019; in Australia, from 9 to 11% between 2014 and 2021; and in Denmark, from 21 to 37% of total crop area between 2019 and 2023 (58). However, other survey evidence also shows substantial variation in adoption of smart farming innovations by crop, for instance, in the United States, 2016 data report automated guidance on 59% of corn hectares, VRT on 50%, yield maps on 45%, and soil maps on 22%, whereas for winter wheat in 2017 automated guidance covered 61% of hectares, VRT 31%, yield maps 15%, and soil maps 4% (59–61).

There is an ongoing debate about the manner and extent to which smart farming innovations can live up to expectations (62, 63). Notably, these expectations diverge: Societal and policy perspectives emphasize sustainability cobenefits, whereas adoption decisions at the farm and agribusiness level are primarily driven by productivity gains, input cost reduction, and risk considerations (57, 62–65). Because of this divergence, the environmental cobenefits of these technologies frequently remain unrealized externalities without strong policy interventions (37, 66). Among the most important variables in this regard are further technological developments (67, 68), countries’ institutions and policies (16, 60), and both farm-level and market-level developments (58, 67, 69). Despite the promise of smart agriculture to enhance productivity and environmental sustainability, global technology diffusion remains low, and even for large companies, it is not clear exactly how the global potential of smart farming is distributed.

To date, most studies rely on one of three approaches: ex-post assessments of technologies already in use, which offer rich insights into realized benefits but cannot predict uptake in new contexts or globally (69–74); small-scale field trials, which test innovations under controlled or localized conditions, yet often fail to capture regional heterogeneity in soil, climate, and especially socioeconomic contexts (74, 75); and ex-ante modeling studies, which use simulations and economic experiments (also choice experiments), based on a mix of theory, data, and assumptions (76–78). Because none of these methods is readily scalable to the global level (either because of the shortage of data or limitations of methods), they leave us with fragmented bits of knowledge of both where smart farming innovations could be most beneficial in terms of profitability and addressing agri-environmental problems, and how to prioritize policies and business choices. To address this gap, we introduce a globally scalable framework to show smart farming innovations’ potential globally—integrating expert surveys, global covariates, and ensemble spatial machine-learning.

We conducted an online survey sent to more than 8,000 selected smart farming experts from private companies, farmers/farmer’s associations, and academia, requesting them to mark locations on a digital global map and complete the following steps: i) select the innovations that have been implemented and are profitable for farmers, ii) click the locations on the digital map where the selected innovations have been used and profitable for farmers, iii) indicate precision of provided coordinates, and iv) indicate certainty about the innovations that have been used in the locations indicated and profitable. The survey was administered via an interactive, browser-based interface accessible on phone, tablet, or computer, through which respondents selected smart farming innovations from a dropdown menu, geo-located profitable deployment sites on an embedded map, and provided assessments of spatial precision and certainty of profitability (*SI Appendix*, Fig. S1). We complemented our survey data with data from a comprehensive meta-analysis (26) (*Materials and Methods*). We incorporated studies that explicitly presented a cost–benefit analysis, revealing the profitability of precision farming technologies among farms/farmers at a spatially explicit level. This provided a large dataset of 4,422 observations, comprising 2,677 (~60%) from our survey and 1,745 (~40%) from the meta-analysis. We include literature that explicitly states the locations and profitability of the specified smart farming innovations. To globally model the potential of each smart farming innovation, we use the data to build an ensemble model of five ML algorithms [Generalized Linear Models (GLM), Random Forests (RF), Support Vector Machines (SVM), Maximum Entropy (MaxEnt), and Boosted Regression Trees (BRT)]. We selected 18 theory-driven predictor variables that have been conceptually and empirically linked to the economic potentials of the innovations (*Materials and Methods* and *SI Appendix*, Table S2) (69).

Our study makes three primary contributions. First, we produce global maps showing the profitability of each smart farming innovation across global croplands. This is crucial information for policymakers and businesses alike, as currently the empirical evidence on this is scattered, patchy, and methodologically not sufficiently unified. Second, we make a first attempt to link the profitability of smart farming innovations to address specific environmental sustainability challenges to the global occurrence of those sustainability challenges. For this, we overlay the profitability of all smart farming innovations with global maps of biodiversity intactness (79), the profitability of autonomous irrigation systems with a map of relative water scarcity (80), the profitability of variable rate technology with agricultural nitrogen pollution (81), and the profitability of automated weeding machines with the risk of pesticide pollution. This helps to identify promising intervention hot spots globally. Third, we integrate the Shared Socioeconomic Pathways (SSPs) framework to project the future adoption potential of smart farming innovations under the SSP2 (also called “Middle of the Road”) scenario for 2035 and 2045, adjusting key socioeconomic and climatic predictors accordingly. We use the SSP2 scenario, which assumes that social, economic, and technological trends do not deviate markedly from historical patterns, and we adjust key socioeconomic and climatic predictors to reflect this intermediate development trajectory (27). Additionally, we simulate the potential of Smart Farming Innovations under contrasting environmental stringency assumptions using the Environmental Performance Index (EPI). We selected EPI for scenario exploration because it emerged as the most important predictor based on mean feature importance across our five ML algorithms (82)—indicating that environmental governance exerts the strongest influence on smart farming adoption potential (*SI Appendix*, Figs. S2, S12, and S13). For the optimistic and pessimistic scenario, we set each country’s EPI to the value of the highest-ranked country within its respective World Bank income group and the lowest-ranked country within each group respectively (83)—thereby capturing realistic regulatory bounds among economically similar countries [World Bank income groups: High income, Upper-middle Income, Lower-middle Income, and Low Income (84)]. Such scenario-based projections are essential for informing anticipatory policy interventions, as current conditions alone cannot guide long-term agricultural planning under evolving socioeconomic and climatic trajectories (27, 85).

Our results below are relevant for farmers, agribusiness professionals, environmentalists, policymakers, and researchers working toward more sustainable agriculture. Additionally, our results are helpful for manufacturers of smart farming equipment that are developing new technologies to streamline agricultural market operations to understand where the potential markets for their products lie—currently and in the future. Policymakers are better positioned to craft effective support and regulations with the new information about smart farming potential globally.

## Global Economic Potential of Smart Farming Innovations.

**Fig. 1.**
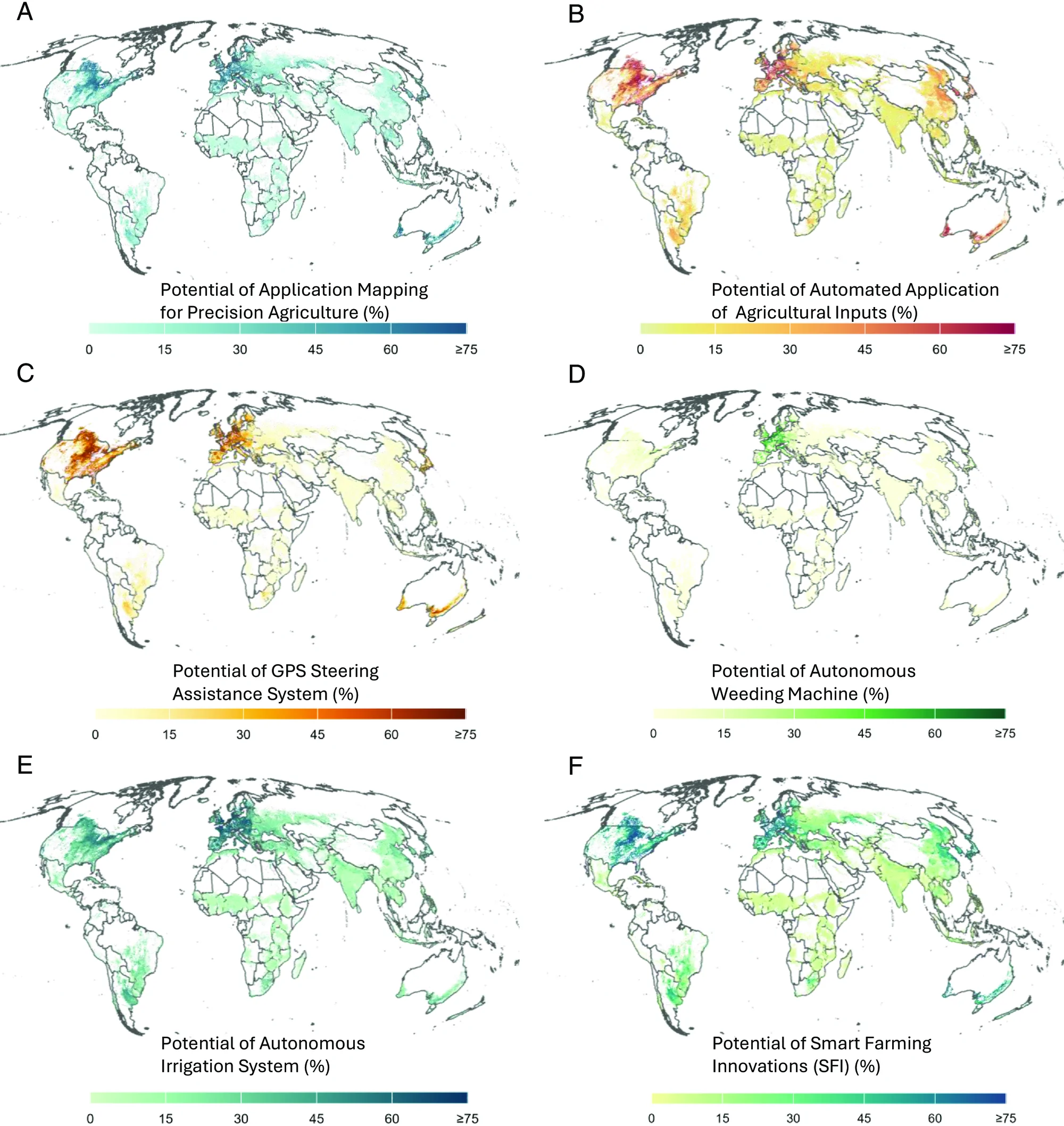
Global potential of smart farming innovations. The maps show the economic potential, indicating the likelihood that a specific technology is profitable for farmers adopting smart farming innovations. Modeled with an ensemble of geospatial ML algorithms at a spatial resolution of 5 km, separately for application mapping for precision agriculture (*A*), automated application of agricultural inputs (*B*), GPS steering assistance (*C*), automated weeding machines (*D*), automated irrigation systems (*E*), and aggregated across all innovations (*F*).

We model the global economic potential of the different smart farming innovations, defined as the probability that an innovation is already profitable for at least some farmers within the given location (at pixel level). Our findings reveal both common patterns and heterogeneities across innovations (Fig. 1). Globally, there is generally a strong correspondence between the innovations’ economic potential and socioeconomic development. As a general pattern, and confirming expectations, smart farming innovations have the most potential in Western Europe, North America, and generally high-income countries such as Australia, Israel, South Korea, and Japan (Fig. 1). In particular, the USA and Europe emerge as the global smart farming hotspots, which is likely explained by a combination of high labor costs, a political and societal priority for sustainable high-value production promoting environmental sustainability, high per-capita income, education, and investments in physical and institutional conditions to support the adoption of smart farming innovations.

To model the economic potential of the smart farming innovations, we relied on five complementary ML algorithms: GLMs, RFs, support vector machines, maximum entropy, and boosted regression trees. For each innovation, we separately computed several performance metrics, which all indicate good model fit (*SI Appendix*, Tables S3 and S5). The most difficult to model innovation was the autonomous irrigation systems, as there are currently not much data available for them. Currently only a small fraction of the world’s irrigated cropland employs sprinkler or drip systems amenable to automation, with the vast majority still relying on furrow or flood irrigation (86, 87). Nevertheless, we included autonomous irrigation systems in this study because escalating water scarcity and increasing demand for water-use efficiency are expected to drive a transition from conventional to precision irrigation methods in the coming decades (30, 31). Individual model outputs were ensembled, leveraging the complementary strengths of each algorithm. To enhance generalization and lower potential model overfitting, we implemented fivefold spatial cross-validation by withholding geographically clustered samples during testing and training. This approach yields more realistic and typically more conservative accuracy estimates by accounting for spatial autocorrelation, compared to relying on random splits (88, 89). We calculated the effective range of spatial autocorrelation—ensuring that folds were spatially independent—to partition the data into five geographically distinct folds, thereby ensuring that the test subsets (~20%) were spatially independent of the training subsets (~80%) across all runs separately. See *Materials and Methods* for the details, as well as a range of checks and analytics (uncertainty *SI Appendix*, Fig. S3 and robustness check *SI Appendix*, Fig. S4 and area of applicability *SI Appendix*, Fig. S5).

## Linking Economic Potential to Global Sustainability Challenges.

**Fig. 2.**
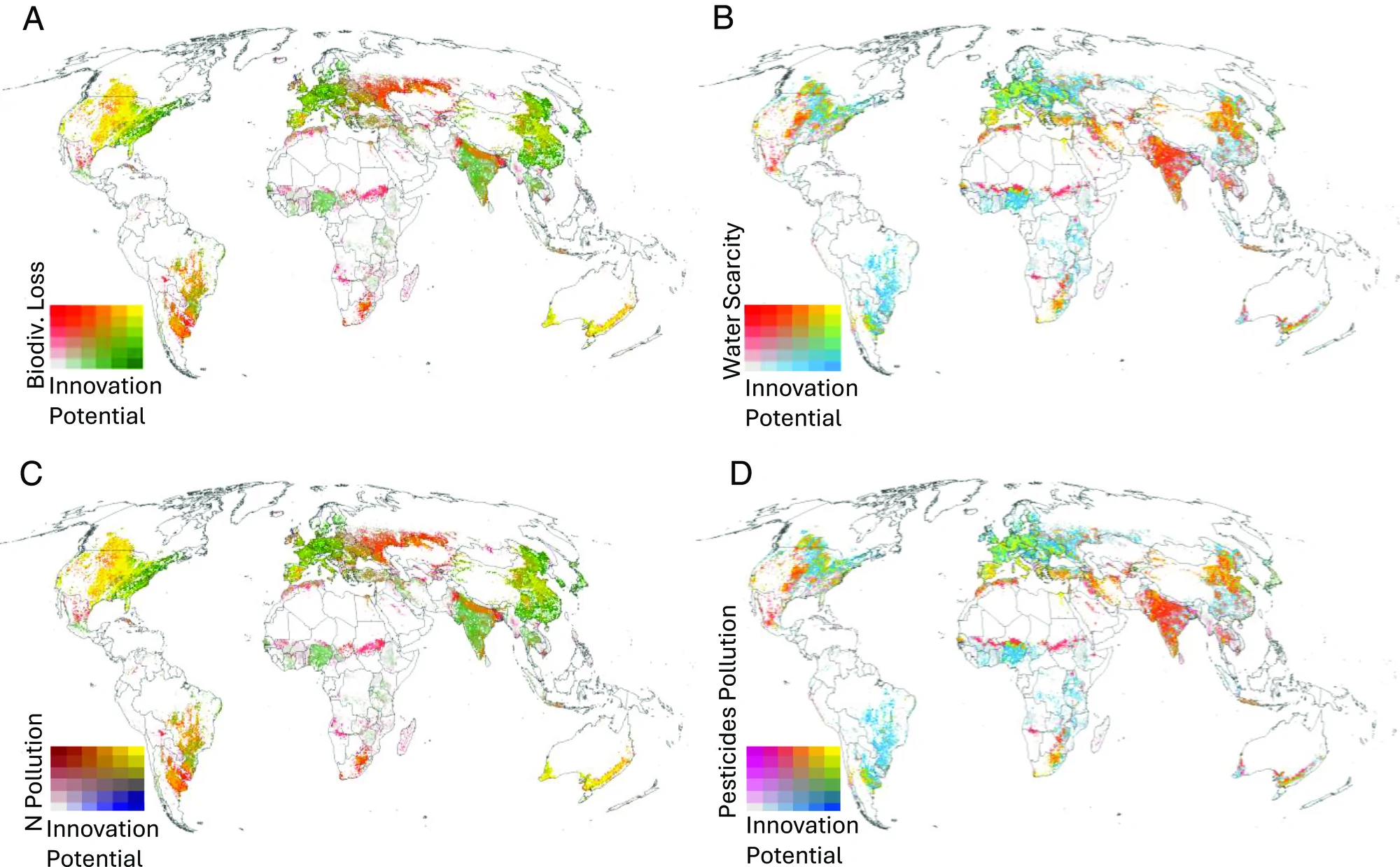
The economic potential of selected smart farming innovations and grand sustainability challenges. For these maps, we matched the aggregate profitability of smart farming innovations with a global map of biodiversity loss (reverse of biodiversity intactness index) (79) (*A*), the profitability of smart irrigation systems and a map of global water risk (80) (*B*), the profitability of variable rate technology and agricultural nitrogen pollution (agricultural nitrogen pollution) (81) (*C*), and the profitability of automated weeding machines and pesticide pollution risk (92) (*D*).

Smart farming innovations can play an important role globally in addressing pressing environmental sustainability challenges, such as biodiversity loss, water stress, and agrochemical pollution (16), given SFI’s demonstrated potential to substantially reduce agricultural input waste and improve resource-use efficiency. This is particularly relevant as nutrient losses to the environment are well-established drivers of biodiversity decline globally (90, 91). Overlaying the economic potential of selected smart farming innovations with these grand sustainability challenges, we see some interesting patterns (Fig. 2). For this analysis, we overlaid the economic potential of all smart farming innovations with a map of the global pattern of biodiversity loss (79) (Fig. 2*A*), the economic potential of automated irrigation systems with a map of global water stress (80) (Fig. 2*B*), the economic potential of automated application of agricultural inputs with a map of fertilizer pollution (agricultural nitrogen pollution) (81) (Fig. 2*C*), and the economic potential of autonomous weeding machines with a map of pesticide pollution (92) (Fig. 2*D*).

Areas with high rates of biodiversity loss and high economic potential of smart farming solutions are especially concentrated in North America, Australia, and are medium in South America, Western Europe, South Africa, and China (Fig. 2*A*). A combination of medium to high smart irrigation economic potential and water stress is found especially in many regions, including North and South America, Europe, and much of Asia (Fig. 2*B*). The economic potential of automated application of agricultural inputs and high degrees of fertilizer pollution come together especially in China, Western Europe, North America, and medium degrees in India and Australia (Fig. 2*C*). A similar pattern is found for autonomous weeding machines’ potential and pesticide pollution—with the visible exceptions being Western Europe, Northern Europe, and the “Corn Belt” of the United States (Fig. 2*D*).

## Projecting Smart Farming Potential under Shared Socioeconomic Pathway 2-4.5 (SSP2) and Environmental Stringency Scenarios for the Years 2035 and 2045.

**Fig. 3.**
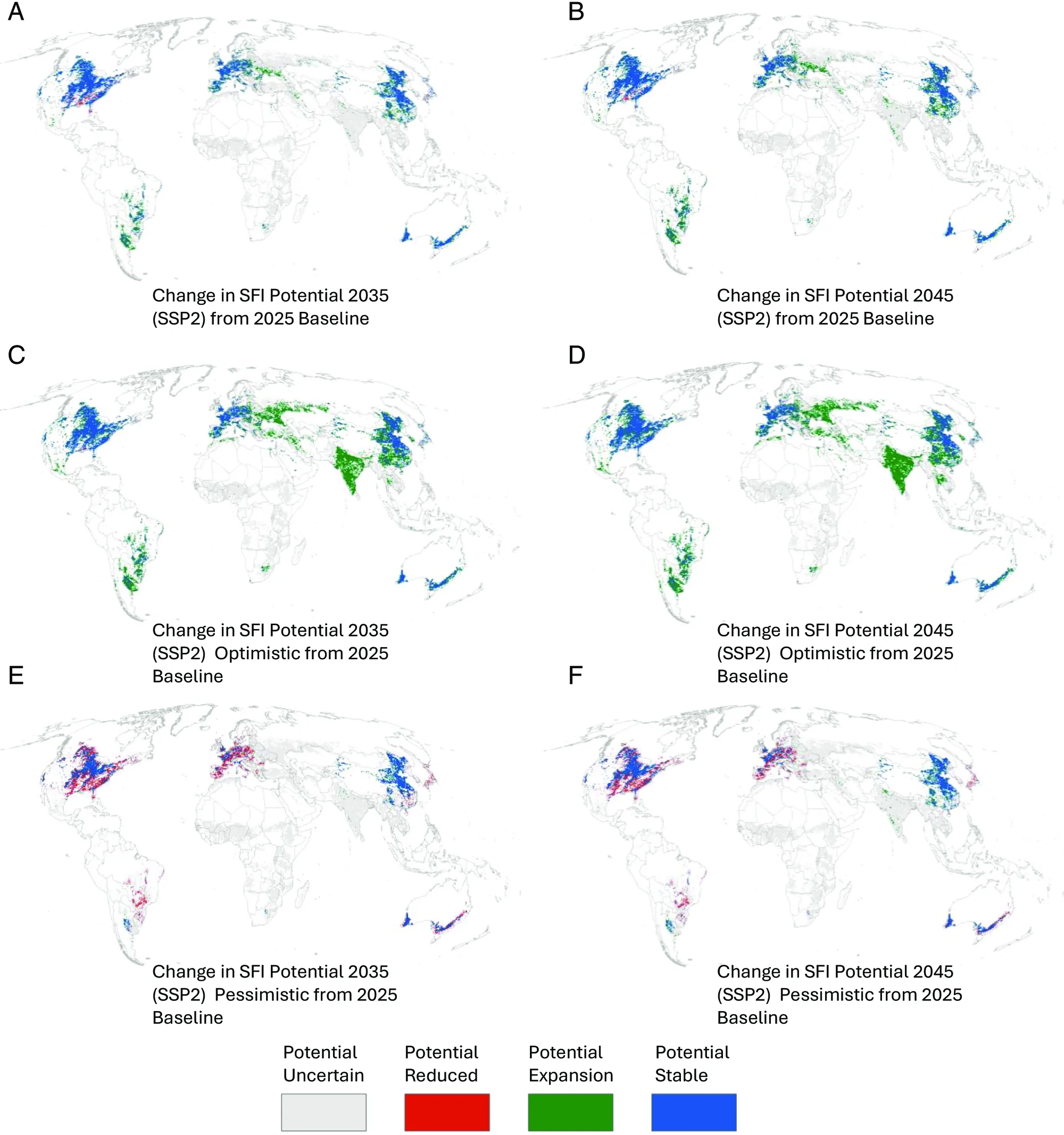
Projected changes in global potential for SFI relative to the 2025 baseline under the SSP2 “Middle of the Road” Pathway with varying environmental stringency (*SI Appendix*, Fig. S10, Panel *G*). (*A*) Change in SFI potential for 2035 under SSP2 baseline assumptions; (*B*) Change in SFI potential for 2045 under SSP2 baseline assumptions; (*C*) Change in SFI potential for 2035 under the optimistic scenario (high environmental stringency), with EPI adjusted to the maximum 2024 value within each country’s World Bank income group (84); (*D*) Change in SFI potential for 2045 under the optimistic scenario; (*E*) Change in SFI potential for 2035 under the pessimistic scenario (low environmental stringency), with EPI adjusted to the minimum 2024 value within each country’s income group; (*F*) Change in SFI potential for 2045 under the pessimistic scenario. Four change categories are mapped relative to the 2025 baseline: Potential Reduced (red), Potential Uncertain (gray), Potential Expansion (green), and Potential Stable (blue).

An important consideration is how the ongoing socioeconomic developments and changes in environmental conditions could shape future changes in the potential of the smart farming innovations (SFI) examined in this study. We report this for the five SFIs combined in order to address the overall objective of the study. We projected the economic potential of SFI for 2035 and 2045, aligned with Shared Socioeconomic Pathway 2 (SSP2-4.5; “Middle of the Road”) and Environmental Stringency, and compared the changes (Fig. 3) relative to the 2025 potentials (*SI Appendix*, Fig. S10, panel *G*). To assess temporal changes in SFI potential, continuous probability surfaces were converted to binary using a threshold of 0.39, determined by maximizing the sum of positive and negative predictive values max(PPV+NPV); PPV = 0.97, NPV = 0.91, which optimizes the trustworthiness of each pixel’s classification on the map. The binarized maps were then compared between the baseline and each scenario to quantify changes in economically viable cropland over time (Fig. 3).

Under the SSP2 baseline scenario, regions with well-established agricultural technology infrastructure—including the U.S. Midwest, Eastern and Central Europe, and major cropping zones in Eastern China and Australia—maintain high smart farming potential at both 2035 (Panel *A*) and 2045 (Panel *B*) (in Blue). Emerging economies (in green) show the greatest gains: By 2035, Eastern Europe, South America (especially Brazil and Argentina), and China reach moderate to high potential, which intensifies further by 2045. Globally, the economic potential of Smart Farming Innovations on total cropland is projected to expand steadily under the baseline pathway, growing from 501.5 million ha (42%) in 2025 to 543.5 million ha (46%) in 2035, and reaching 588.5 million ha (50%) by 2045.

Under the High Environmental Stringency (“optimistic”) scenario—where each country’s EPI is set to its income-group maximum—smart farming potential on total cropland area expands to 839.2 Mha (~71%) in 2035 and 886.3 Mha (~75%) in 2045 (Panels *C* and *D*). In contrast, the Low Environmental Stringency (“pessimistic”) scenario—with each EPI at its income-group minimum—yields only 322.0 Mha (~27%) in 2035 (reduced by 179.5 Mha; –35.8%) and 373.1 Mha (~31%) in 2045 (reduced by 128.4 Mha; –25.6%). The optimistic–pessimistic gap remains around 517 Mha in 2035 and 513 Mha in 2045.

Notably, the comparison between the optimistic scenario for 2035 (Panel *C*) and the baseline SSP2 projection for 2045 (Panel *B*) reveals a striking finding: Regions such as Eastern Europe, South America, China, India, and Thailand exhibit higher smart farming potential under the 2035 optimistic scenario than under the 2045 baseline SSP2 projection, in total, 50 to 71% increase of the smart farming potential on global cropland. This underlines the pivotal role of environmental governance and policy in shaping the potential of smart farming technologies. Despite projected gains in several developing regions, pronounced geographic disparities persist across all scenarios. Sub-Saharan Africa consistently displays low potential in smart farming innovation (SFI) adoption—even under the optimistic projections for 2035 and 2045 (gray, Fig. 3). Central Asia and parts of the Middle East likewise exhibit only modest expansion over time. (“Potential” refers to the modeled probability of smart farming being feasible and profitable for the farmers in a given region).

## Discussion

We find that smart farming innovations have considerable potential across the globe to make agriculture more productive, resilient, and sustainable. This potential is especially high in North America and Western Europe, where diffusion is already relatively more advanced, and early experiences show that they can reduce input costs and boost yields (93). The potential of some innovations is globally quite broad, whereas the potential of other innovations is currently much more geographically concentrated. An example for the latter are weeding robots, which currently mostly show potential in Western Europe, and there mostly for herbicide-intensive row crops (e.g., sugar beets) (94). While herbicide-resistant weeds create demand for similar solutions in the United States, European-designed weeding robots are currently not designed for the needs of large US commodity farms. Consequently, U.S. farmers are currently rather adopting machine-vision smart sprayer technologies for targeted herbicide application, which can operate efficiently at very large scales for addressing herbicide resistance (95, 96). However, all the considered innovations are also constantly being improved and farmers and companies learn how to use and integrate them better. An example is the combination of automated weeding robots with AI for disease identification and biological remedies, allowing organic production to produce more at lower cost (36).

Compared to Western Europe, Eastern Europe shows generally lower potential for smart farming innovations currently, which is largely explained by wider economic differences between the regions. However, Eastern European economies are growing, and in our analyses of future innovation dynamics (Fig. 3), we see that the most potential for expansion lies in middle-income countries in Asia, South America, and Eastern Europe. These are regions that account for a considerable share of the major environmental challenges considered above (Fig. 2). For example, many countries in South and East Asia face significant fertilizer and pesticide pollution, as well as water scarcity. With future expansion of the economic potential of smart innovations, this will considerably increase the overlap we currently observe (Fig. 2).

This macroeconomic trajectory has direct implications for farm-level adoption choices. As evidenced by microeconomic behavioral experiments and agricultural adoption literature, technology diffusion is heavily constrained by upfront capital costs, risk aversion, and technical skill deficits (69, 97, 98). Smart farming technologies are especially “human-capital intensive” and require substantial initial investment (53, 99). As regional socioeconomic conditions, education, environmental performance, and institutional support frameworks improve, farmers are better equipped to overcome these financial and cognitive barriers (97, 99), thereby translating theoretical regional profitability into actual, farm-level adoption decisions.

Our findings also reveal regional disparities in the economic potential of smart farming innovations, with sub-Saharan Africa and parts of South Asia consistently exhibiting lower potential even in optimistic environmental governance scenarios. These patterns align with the broader discourse on agricultural technology transfer and the view that “inappropriate technology” is a globally meaningful technology diffusion barrier. Agricultural technologies are predominantly developed in and for high-income country contexts, resulting in innovations that may be ill-suited to the agroecological, socioeconomic, and institutional conditions elsewhere (100). This technological mismatch—wherein e.g., precision agriculture tools are designed for large-scale, capital-intensive farming systems prevalent in North America and Western Europe—may partly explain the persistently lower potential observed in structurally vastly different regions in our projections. Consequently, our findings primarily speak to the adoption potential of technologies designed for these production systems. Smart farming technologies developed specifically for other agroecological and socioeconomic contexts may exhibit different adoption dynamics. Our scenario analysis further underscores this: While improvements in environmental governance (optimistic scenario) can accelerate adoption readiness in emerging economies such as China, India, and Brazil, the gains remain limited in the lowest-income regions, suggesting that socioeconomic development and regulatory improvements alone may be insufficient. Addressing this gap requires not only investments in infrastructure and human capital but also a fundamental reorientation of agricultural research and development toward context-appropriate innovations tailored to smallholder farming systems, diverse agroecology, and similar environments, for example, in India and China (100, 101). Thus, new technologies emerging from structurally more similar countries to those common in Africa and South Asia can be expected to have greater potential in the regions currently showing low economic potential for most smart farming innovations. This mirrors the patterns recently found for entrepreneurship, where entrepreneurship diffuses more easily and faster among structurally more similar countries (101).

However, across all simulations, we found low smart farming adoption potential in sub-Saharan Africa, yet, there are some optimistic projections that emphasize how digital agriculture projects also in sub-Saharan Africa already enhance market access and productivity for participating farmers (33). Previous studies have found considerable growth in drone use for spraying fertilizer or pesticides in Africa and Southeast Asia. This, however, is usually not combined with precision application, but rather continues to be used with fixed rates for entire fields. Nevertheless, there are both positive and negative implications for the environmental sustainability of these farms (102, 103). Whereas our results suggest that the potential of the different smart farming innovations is unclear (rather low), e.g., in sub-Saharan Africa. Ultimately, this is truly difficult to predict, as the smart farming potential in sub-Saharan Africa depends not only on economic growth but also on institutional and policy changes, alongside value chain development, and the adaptation of the required technologies.

In many regions of the world, there is considerable potential for smart farming innovations, and this can be expected to contribute to a more efficient, sustainable, and productive use of agricultural inputs. We find several regions globally where one can make an economic case for the support of smart farming to mitigate biodiversity loss, and pesticide and nitrogen pollution—particularly in North America, Western Europe, Australia, as well as in China.

With continuous socioeconomic development, these technologies can further reduce environmental impacts in South America, Eastern Europe, and parts of Asia. Beyond economic development, it should be noted that there is also considerable technological development happening around the globe [e.g., in large research clusters such as PhenoRob (104), AIFARMS (105), patchCROP (106, 107), or Hands Free Hectare (108, 109)]. Technological advancements have already made these innovations cheaper and more convenient to use, and this can be expected to continue, as smart farming is still in an early diffusion phase and there is broad willingness to continue investing in them (22, 110).

At the same time, important adoption barriers exist, and there are future risks as well. This includes microeconomic barriers, including human and financial capital constraints, risk aversion on the side of farmers, companies, and policymakers alike, as well as path dependencies that can lock in agricultural systems over time, and there can be strategic considerations, with farmers waiting for the optimal moment when to switch to new practices (16, 111). Taxes on inputs such as fertilizer and pesticides, result-based agri-environmental payments, targeted public and private intervention (including subsidies), infrastructure investments, and policy reforms, all can promote the adoption of these innovations (16).

Given our overall findings regarding the potential of smart farming innovations—both economically and their spatial alignment with critical sustainability challenges, policymakers might consider integrating smart farming into national strategies—leveraging subsidies, extension services, and infrastructure investments—to bridge the digital divide and unlock socioeconomic benefits, as advocated in the World Bank’s “Digital Future of Food and Agriculture” roadmap (112).

Continued research efforts are necessary, particularly within inter- and transdisciplinary frameworks, examining the intertwined barriers and drivers of smart farming adoption, carefully assessing long-term impacts and feedback loops, and informing the design of context-sensitive interventions (e.g., location-tailored, targeted training programs, risk-sharing mechanisms, and connectivity initiatives). A particularly important aim should be how to best align innovation incentives with sustainability, equity, and capacity-building objectives, so that stakeholders can ensure that smart farming not only furthers economic objectives but, in particular, advances broader societal and environmental goals. An alternative opportunity for future research would be to bring together the expected future diffusion of smart farming innovations with the expected dynamics of environmental challenges. This would allow a forward-looking assessment where in the world we might expect future overlap between the economic potential of smart farming innovations and their potential to mitigate environmental issues.

Another future research need is the continuation of systematically and jointly analyzing the technological and the economic aspects of smart farming, in order to understand both how to support wide diffusion and societal benefits, as well as to anticipate risks and issues early on (113). There is, e.g., both potential for under- and for overregulation. With too little regulation, smart farming innovations might be used suboptimally from a societal perspective, and smart farming innovations can even be used in ways that could increase environmental and health costs instead of reducing them. On the flip side, the economic potential of smart innovations depends strongly on how much they are regulated, and with a high administrative and bureaucratic burden, and many safety precautions, innovations such as automated machinery might lose any economic advantage they might otherwise have. Thus, smart farming is an interesting case for cost–benefit analyses that can inform policies that strike a balance between economic freedom and entrepreneurship, and safeguard society from downsides of technological progress.

## Materials and Methods

This section outlines the methodologies employed to model the economic potential of smart farming innovations. It contextualizes the selected key technologies, the collection and processing of data, the development and evaluation of geospatial ML models, and the techniques used for scenario simulation. A comprehensive overview of the complete methodological workflow, including main steps, data sources, and robustness checks, is provided in *SI Appendix*, Fig. S6.

### Smart Farming Technologies Investigated.

We selected five broad categories of smart farming innovations based on three criteria: 1) potential for input reduction and efficiency gains (57, 114), 2) documented or expected environmental cobenefits (115), and 3) current market availability and commercial maturity (i.e., technologies beyond the experimental stage) (*SI Appendix*, Table S4). Livestock technologies, robotic harvesting, farm management software, vertical farming, and blockchain systems, for example, were excluded—because their adoption is governed by different determinants (livestock density, crop-specific, digital infrastructure, market access) that fall outside our spatially explicit, crop-focused modeling framework.

Finally, selected smart farming innovations are as follows:

1. Application Mapping for Precision Agriculture: This involves remote sensing technologies (e.g., drones and satellites) used to produce application guide maps for agricultural fields, enabling the precise application of fertilizers, pesticides, and other inputs tailored to specific soil and crop conditions.
2. Automated Application of Pesticides, Fertilizer, or Other Inputs: This category includes application machines that precisely (semi-) autonomously apply agricultural inputs based on application maps (e.g., drones, robots, etc.).
3. Autonomous Weeding Machines: These machines are equipped with AI and/or (semi-) autonomous navigation capabilities, designed to identify and remove weeds in agricultural fields with minimal human intervention.
4. GPS Steering Assistance Systems: This technology involves Global Positioning System-based auto-steer systems for agricultural machines, which allow for more precise driving paths, resulting in fuel saving, reduced input use, and minimized soil impact.
5. Autonomous Irrigation Systems: These precision irrigation systems utilize sensors to adjust irrigation rates according to actual soil and plant needs, contributing to efficient water resource management.

### Training Data.

We undertook an extensive global survey from November 2023 to August 2024 to get the geolocations where the smart farming systems are adopted and profitable. We employed a nonprobability, purposive sampling strategy (23, 24), to identify key informants with specialized knowledge of smart farming technologies. We reached more than 8,000 experts, including farmers, researchers, and companies that either use, study, or sell innovative smart farming products globally. The experts were selected from three distinct informant groups: 1) end-users—farmers and farmers’ associations with confirmed technology adoption; 2) industry leaders—CEOs and senior officials of companies manufacturing smart farming hardware and software; and 3) academic experts—researchers with published empirical studies on the relevant technologies. Expert identification proceeded through four complementary channels; first, trade fair engagement: We attended AGRITECHNICA 2023 (Hanover, Germany), the world’s leading agricultural technology trade fair. We directly engaged exhibitors showcasing relevant smart farming innovations and distributed a survey leaflet (*SI Appendix*, Fig. S7) to exhibitors and attendees (specifically farmers who already adopted similar technologies), which was also distributed at the ICABR 2024 conference among researchers. Second, we identified smart farming technology providers through web searches and industry databases, contacting company leadership and regional distributors via email. Third, we contacted researchers at universities and research organizations actively working on listed technologies (e.g., PhenoRob, AIFARMS). Here, we employed snowball sampling (25), whereby initial respondents from all three informant groups were requested to recommend other qualified experts within their professional networks, thereby expanding the reach of our survey beyond the initially identified contacts and helping to capture expertise in regions and technologies that were otherwise underrepresented. Finally, we incorporated georeferenced adoption data from Schulz and Börner (26), a comprehensive meta-analysis of agricultural technology adoption studies, obtained directly from the corresponding author. No publication bias was reported in that analysis.

Our final dataset comprises 4,422 responses from 67 countries (*SI Appendix*, Fig. S8), from 30 companies that produce smart farming technologies (n = 453, ~11%), 28 research organizations and universities (n = 2,047, ~46%), meta-analysis extraction (n = 1,745, ~40%), and direct farmer responses (n = 177, ~4%). The largest numbers of points were recorded in the United States (n = 855), France (n = 706), Canada (n = 693), Australia (n = 596), Netherlands (n = 337), and Germany (n = 211) (*SI Appendix*, Fig. S9).

Responses came primarily from experts in academia and research organizations, while feedback from farmers or farmers’ organizations was the least represented. Due to our use of a snowball survey approach, along with email outreach and leaflets, we were unable to assess any systematic bias between respondents and nonrespondents. However, to assess the relationship between respondent typology and reported certainty (following question iii), we conducted a cross-tabulation with a simulated Chi-square test of independence, which revealed a highly significant association (*P* < 0.001, X^2^ = 4,767.3). The pattern is substantively meaningful: Commercial entities (Companies) exhibited the strongest confidence, with 40.2% rating their assessments as “very certain” and 21.6% as “completely certain.” Researchers and University experts showed a similar pattern, with nearly half of the responses categorized as “highly certain” (49.1%), and a substantial proportion as “very certain” (44.9%). By contrast, Literature Reviews were predominantly “fairly certain” (69.2%), and Farmers were the only group to express notable uncertainty (15.8%).

Participation was self-administered through the EAT-Database Survey Shiny application. We posed four ordered data-collection items to them, including:

i. From the innovations listed below, please select the innovations that have been adopted and are profitable for farmers in specific locations.
ii. Click the locations on the map where the selected innovations have been adopted and are profitable for farmers.
iii. What is the approximate precision of your provided coordinates?
iv. How certain are you that your selected innovation has actually been already adopted in the locations you indicated and that this was profitable? See (*SI Appendix*, Fig. S1); the complete verbatim instrument, including response options and interface instructions, is provided in *SI Appendix*, Table S6. (*All responses were collected by the authors through their own application without any external survey company or commercial panel, and no AI or synthetic respondents were used at any stage of the survey workflow*).

To supplement survey data, we also incorporated data from a meta-analysis study “Innovation Context and Technology Traits Explain Heterogeneity Across Studies of Agricultural Technology Adoption: A Meta-analysis” (26).

In total, 5,679 geolocations were initially collected from both survey and meta-analysis. After a comprehensive data cleaning process, which involved dropping duplicates, uncertain points, and points not located on cropland, we compiled a total of 4,422 unique coordinates for all innovations combined (*SI Appendix*, Table S1). For the potential of smart farming innovations, these points were spatially thinned to a 5 km pixel level, further reducing the number to 2,077 for better representativeness. However, for modeling individual SFI innovations (Nos. 1 to 5), specific training points were utilized for each Smart Farming Innovation (*SI Appendix*, Table S1).

For each smart farming innovation, we generated the background points from 150 countries following best practices (116). From the survey and meta-analysis, we obtained different numbers of presence points for different countries. Therefore, we followed a dynamically weighted approach in deriving the number of background points per country to ensure balanced representation. First, we restricted the number of background points to range between 10 and 50 per country. However, for countries with less than 50 training points, we picked an equal number of background points and presence points (117). Within each country, we randomly sampled background points so that their number did not exceed the number of recorded smart farming innovation geolocations. This “one‐to‐one” ratio prevents any single country from disproportionately influencing model outcomes and maintains a balanced representation of the samples. By including all cropland areas—rather than restricting background points to regions adjacent to known smart farming innovation geolocations—we captured the diversity of environmental and socioeconomic contexts in which each innovation might be feasible. Another reason is that we have five important country-level predictors (e.g., EPI, Investment Freedom Index, Government Spending Index, Property Rights Protection Index, Economic Freedom Index), enabling us to capture the heterogeneity among all countries more accurately. This approach minimizes geographic sampling bias and helps the models distinguish true probability signals from broad regional differences in cropland availability and data coverage.

### Predictors.

We compiled 18 covariates hypothesized to influence the economic potential of smart farming innovation (*SI Appendix*, Table S2) grouped as socioeconomic, infrastructure, environmental, agronomic, and national indices. The socioeconomic factors included the Economic Freedom Index (118), and the Human Development Index (HDI) (119). We employed the HDI rather than per capita income as a socioeconomic predictor, as HDI simultaneously captures three dimensions critical to smart farming adoption: financial capital (income), human capital (education), and health capital (life expectancy). While income is an important determinant of technology adoption, education level and public awareness of environmental and health concerns are equally influential drivers of smart farming uptake (69, 97, 120). Property Rights Protection (28) served as a form of governance indicator. The infrastructural variable was represented by travel time to cities (121). Environmental variables included soil organic carbon and soil water content at approximately 5 km grid resolution (122); warm-quarter precipitation and temperature at a ~5 km resolution (123); terrain ruggedness and mean elevation at 250 m resolution (124). The agronomic predictors included the number of crop types (125), cropland fraction at a 3 km resolution (126), demographics (127), and field-size classification (128).

Furthermore, three additional national indices—Environmental Performance (129), Government Spending (28), and Investment Freedom index (28)—were included for the year 2023. Night-time Lights (130) were also included; derived from satellite imagery, they serve as a proxy for broader socioeconomic development, infrastructure availability, and electrification (131, 132). Most continuous predictor variables were resampled to 5 km resolution using the bilinear interpolation method, except for population demography (working-age population from ages 15 to 64), which was resampled using the sum method. For the Temperature of the Warmest Quarter, Precipitation of the Warmest Quarter, and Cropland Area, we applied the mean method. In contrast, the categorical variable was resampled using the nearest neighbor method to maintain the integrity of the categories.

### Model Fitting.

For each smart farming innovation category, we trained five geospatial ML models—GLM, RF, SVM, MaxEnt, and BRT, using the sdm package (v.1.2.56) (133) in R (v.4.4.2) (134), where sdm uses default hyperparameter settings for each model from its source package (*SI Appendix*, Table S3). All models were fitted with the same set of 18 covariates (*SI Appendix*, Table S2). We split the dataset into training and testing sets across five geographically defined folds to ensure spatial independence using the blockCV package (89). The model was iterated five times for each algorithm (n = 5). Blocking distances were defined based on mean interpoint separations so that training and test subsets within each fold were spatially independent. We evaluated performance using the area under the ROC curve (AUC), Pearson’s correlation coefficient (COR), True Skill Statistic (TSS), and model deviance.

For SFI, all five algorithms achieved uniformly high predictive performance (AUC > 0.92; *SI Appendix*, Fig. S11 and Table S5), yet showed differences in performance level. The GLM attained an AUC of 0.929 ± 0.018 and a Pearson’s COR of 0.779 ± 0.040—a result that is proof: a linear model achieving this level of spatial discrimination on genuinely held-out partitions confirms that our 18 predictors collectively form a well-specified, near-linear signal for smart farming adoption potential, directly validating the predictor selection process. Nevertheless, the nonlinear RF consistently and substantially outperformed all other algorithms (AUC = 0.985 ± 0.006; COR = 0.877 ± 0.026; Deviance = 0.510 ± 0.057), with the lowest variance across spatial folds of any method—indicating not only the highest discrimination but also the most stable and well-calibrated predictions. The persistent margin over the GLM implies significant residual nonlinear structure in the data—including threshold effects and complex interactions among governance, infrastructure, and agro-climatic variables—that tree-based ensemble methods are well suited to exploit (135). SVM and MaxEnt achieved intermediate and comparable discrimination (AUC ≈ 0.94), while BRT performed similarly to the GLM in AUC and TSS.

Predictor importance was estimated via a model-agnostic permutation procedure which averages the relative contribution of each variable across all algorithms in the ensemble and is therefore robust to any single algorithm’s idiosyncrasies (*SI Appendix*, Fig. S2). Also, per-algorithm predictor importance shows a similar pattern (see *SI Appendix*, Fig. S12 and heatmap *SI Appendix*, Fig. S13).

### Future Projection Simulation under SSP2 (Middle of the Road Scenario) and Environmental Stringency.

To explore how socioeconomic change might alter smart farming potential, we simulated future scenarios for the years 2035 and 2045 based on the Shared Socioeconomic Pathway 2 (SSP2; “Middle of the Road”). To account for uncertainty in environmental governance, we modeled future potential under two contrasting regulatory pathways by adjusting the EPI. In the “Low Environmental Stringency” (pessimistic) scenario, national EPI scores were set to the minimum current value within their World Bank income group, while the “High Environmental Stringency” (optimistic) scenario used the maximum value. This approach integrates projected socioeconomic development with varying levels of environmental performance while holding other covariates constant.

We trained an ensemble of five ML models using the baseline parameterization and training occurrences, utilizing twelve key predictors: cropland area, population density, EPI, field size, HDI, mean precipitation, nighttime lights, crop diversity, mean temperature, soil organic carbon, soil water content, and travel time to the closest city. To project smart farming potential for 2035 and 2045, we incorporated dynamic projections (adjusted for the relevant years) for HDI (136), population density (27), and climatic variables (137) according to the SSP2 framework. We kept other variables—specifically, field size, cropland area, nighttime lights, crop diversity, soil organic carbon, soil water content, and travel time—that either lack reliable future projections or are expected to remain relatively stable in the near term—static at current levels. All five ML models demonstrated strong performance when evaluated against independent test dataset (AUC: 0.92 to 0.98; cBoyce: 0.82 to 0.96; TSS: 0.72 to 0.87; COR: 0.75 to 0.87), with RF achieving the highest discrimination across all metrics (AUC = 0.98, TSS = 0.87). An AUC-weighted ensemble of all five models was used for final predictions and projections for all scenarios. Binary SFI potential maps were generated using a threshold of 0.39, determined by maximizing the sum of positive and negative predictive values (max(PPV+NPV); PPV = 0.97, NPV = 0.91, ensuring high reliability of spatial predictions for both presence and absence of SFI potential (138).

When projecting how these future scenarios alter the economic potential of smart farming innovations, it is crucial to know where those projections remain reliable. That is why we first delineate the model’s “area of applicability”—the combination of all conditions (variables) for which the algorithm was trained and shown to perform well. With an area of applicability close to 1 (range 0.71 to 1) (*SI Appendix*, Fig. S5) across almost all of the global cropland, we reliably transfer our model to make predictions on the simulated data.

### Ensemble Mapping.

Predicted continuous suitability surfaces from each algorithm were combined using ensemble methods (AUC weighted average) to leverage the complementary strengths among methods (139).

### Overlaying Map.

To reveal where smart farming potential and sustainability challenge mitigation coincide, we produced a series of bivariate maps at a 5 km resolution by combining two continuous model outputs—1) the economic potential of selected smart farming innovations and 2) the overlay with a given environmental sustainability challenge—into a single graphic following best practice (15). Each raster was first divided into six quantile‐based classes to ensure an even distribution of values across global croplands. We then crossed these classes into a 6 × 6 grid of 36 unique bivariate categories, assigning each a distinct color drawn from two divergent sequential palettes. This approach follows established best practices for geospatial visualization and allows readers to instantly identify hotspots where both the smart farming innovation’s potential and the environmental sustainability challenge intensity are high (or low). We applied this method to four innovation–challenge pairings: aggregate smart farming potential with biodiversity loss (79), smart irrigation potential with water stress (80), variable rate technology potential with nitrogen fertilizer pollution (81), and (semi-) automated weeding machine potential with pesticide pollution (92). The resulting maps highlight regions where implementing these technologies could yield dual benefits for productivity and environmental sustainability.

To assess spatial overlap under future socioeconomic and regulatory conditions, we extended this bivariate analysis to the year 2035 using the Shared Socio-economic Pathway 2 (SSP2) “Middle of the Road” framework. We modeled future potential under two contrasting regulatory scenarios to capture the sensitivity of technology adoption to the EPI. In the “Low Environmental Stringency” (pessimistic) scenario, the EPI for each country was adjusted to the minimum value currently observed within its World Bank income group. Conversely, in the “High Environmental Stringency” (optimistic) scenario, the EPI was adjusted to the maximum value within the respective income group.

We then applied the same bivariate mapping procedure described above to overlay these simulated 2035 economic potential layers with the environmental stressor layers. For this temporal analysis, the environmental risk layers (biodiversity loss, water scarcity, nitrogen pollution, and pesticide pollution) were held constant at their current distributions because no pixel-level future projections were available. Consequently, we restricted this specific analysis to the near-future (2035) horizon.

### Robustness Check of Data Sources.

To mitigate potential biases from sparse training observations in regions like sub-Saharan Africa, our framework employs global background point generation, an ensemble of five ML algorithms, and rigorous fivefold spatial block cross-validation. This methodology ensures that predictions are driven by underlying socioeconomic and environmental covariates rather than mere geographic interpolation, allowing the model to generalize reliably to data-poor areas (140). Nonetheless, broad spatial coverage in training data remains important—not because the model spatially interpolates, but because geographically diverse training observations help the model learn the full spectrum of environmental conditions under which smart farming is profitable (117). In this regard, ensuring spatial representativeness of training data is likely more influential than the type of source providing the assessments, as the geographical distribution of training data influences how comprehensively the model captures the environmental conditions associated with smart farming profitability. Nevertheless, to ensure full transparency, the inherently higher local prediction uncertainty associated with these data-sparse regions and with modeling algorithms is explicitly quantified and reported in our supplementary uncertainty maps in *SI Appendix*, Fig. S3.

The SD (uncertainty) of predictions across the five distinct statistical and ML algorithms in our ensemble is shown in *SI Appendix*, Fig. S3. While the overall predictive accuracy of the ensemble is highly robust (AUC > 0.81 across all innovations), localized areas of high uncertainty indicate algorithmic divergence—where parametric (e.g., GLM) and nonparametric (e.g., RF, BRT) models yield differing potential probabilities, typically in regions with sparse training observations or novel environmental conditions.

For the purpose of assessing the robustness of these diverse data sources and their influence on model outcomes, we categorized the survey data into two subsets: data collected solely from researchers and data collected from other sources, specifically farmers and agricultural technology companies. To check the similarity and evaluate the impact of these data origins, we developed three distinct models, presented as maps in *SI Appendix*, Fig. S4: Potential of Smart Farming Innovations and Uncertainty (All data) (Panels *A* and *B*), Potential of Smart Farming Innovations (Researcher) (Panels *C* and *D*), and Potential of Smart Farming Innovations (Except Researcher) (Panels *E* and *F*). The robustness of the data sources was checked through the development of several maps, each utilizing distinct subsets of the collected data.

Specifically, in *SI Appendix*, Fig. S4, Panel *A* illustrates a map generated exclusively from all data, encompassing 4,422 data points. To assess the consistency and variability of this map, Panel *B* presents the SD of Panel *A*’s results across all models and validations. Further refining the analysis, Panel *C* features a map derived solely from data collected by researchers from the survey (2,047 data points), with its corresponding SD shown in Panel *D*. Finally, Panel *E* showcases a map constructed using data collected from farmers and agricultural technology companies (except from researchers), totaling 2,375 data points, and Panel *F* displays the SD for Panel *E*.

### Ethics.

The study protocol was approved by the Research Ethics Board of the Center for Development Research (ZEF), University of Bonn (registration code: “33_Shahrear Roman_23”). All participants gave prior informed consent by acknowledging an information screen describing the survey protocol (*SI Appendix*, Table S6).

## Supplementary Material

Appendix 01 (PDF)

## Data Availability

All data, code, and model outputs required to reproduce the results of this study are archived in the Zenodo repository and are publicly available. The replication package—including privacy-preserving training data, ensemble prediction rasters at 5 km global resolution, scenario projections, and all R analysis scripts—are archived on Zenodo (141). All other data are included in the manuscript and/or *SI Appendix*.
