# Supplementary material for: Modeling the global potential of smart farming innovations
Source: Proc Natl Acad Sci U S A. 2026 Oct 19;123(43):e2530081123. doi: 10.1073/pnas.2530081123 (PMC13715759; doi:10.1073/pnas.2530081123)
Supplement: Supplementary file 1 [file pnas.2530081123.sapp.pdf]

Supporting Information for
Modelling the Global Potential of Smart Farming Innovations

Shahrear Roman[1,4], Hadi Hadi[1], Wyclife Agumba Oluoch[1], David Zilberman*[2], Robert Finger[3], David Wuepper[1]

Affiliations:

[1]Land Economics Group, Institute for Food and Resource Economics, University of Bonn, Bonn, Germany

[2]Department of Agricultural and Resource Economics, University of California, Berkeley, USA

[3]Agricultural Economics and Policy Group, ETH Zürich, Zürich, Switzerland

*David Zilberman
[4]Co-corresponding author

To whom correspondence may be addressed. Email: zilber11@berkeley.edu or roman@uni-bonn.de

This PDF file includes:

Figures S1 to S13
Tables S1 to S6

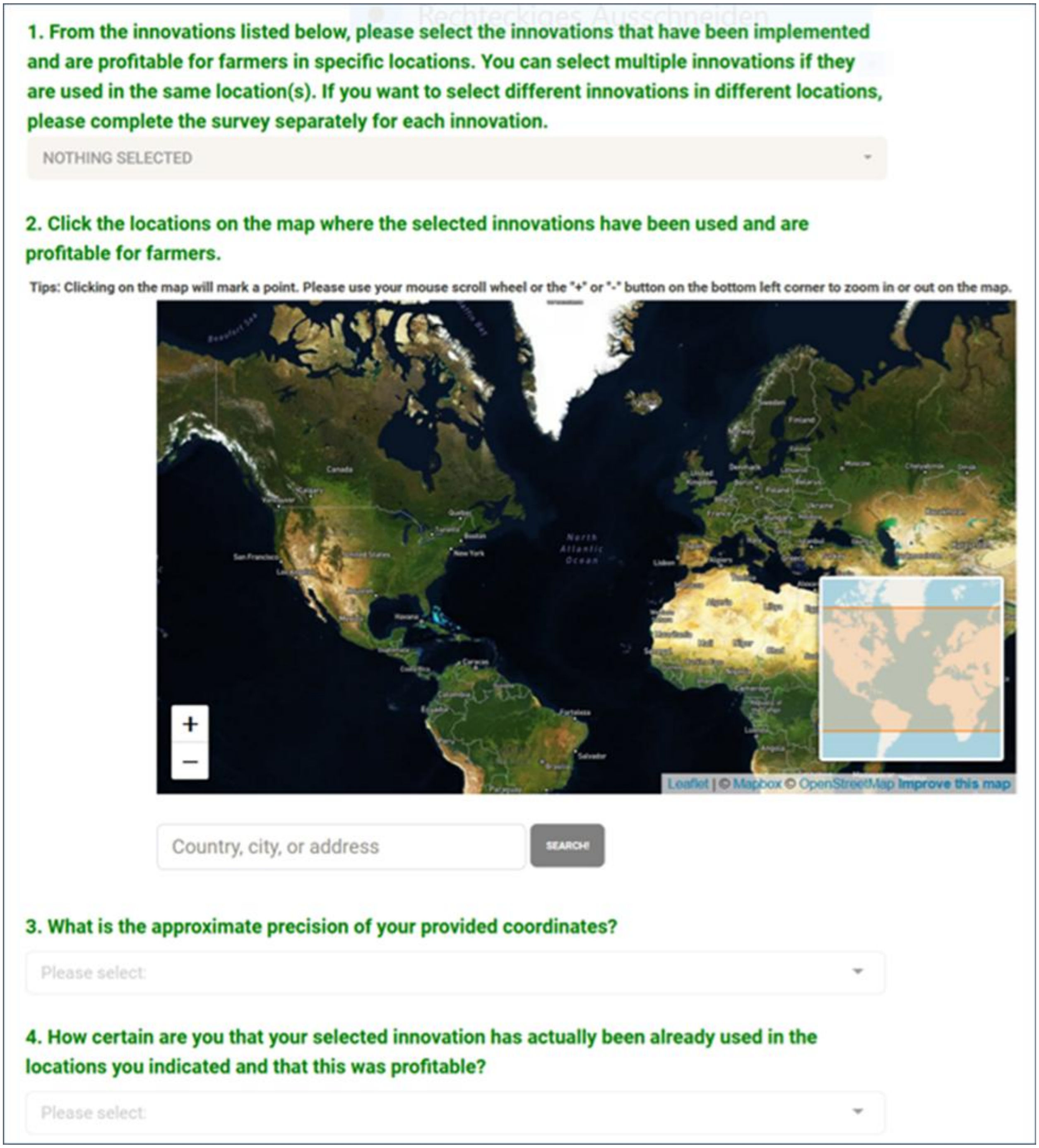

Fig. S1: Interactive, app-style survey interface. This survey is designed for use on phone, tablet, or computer. Respondents first choose one or more smart-farming innovations from a dropdown menu (1), then zoom and pan the field location on the embedded map to pinpoint locations where those innovations have been profitably deployed (2), followed by two more questions regarding precision and certainty.

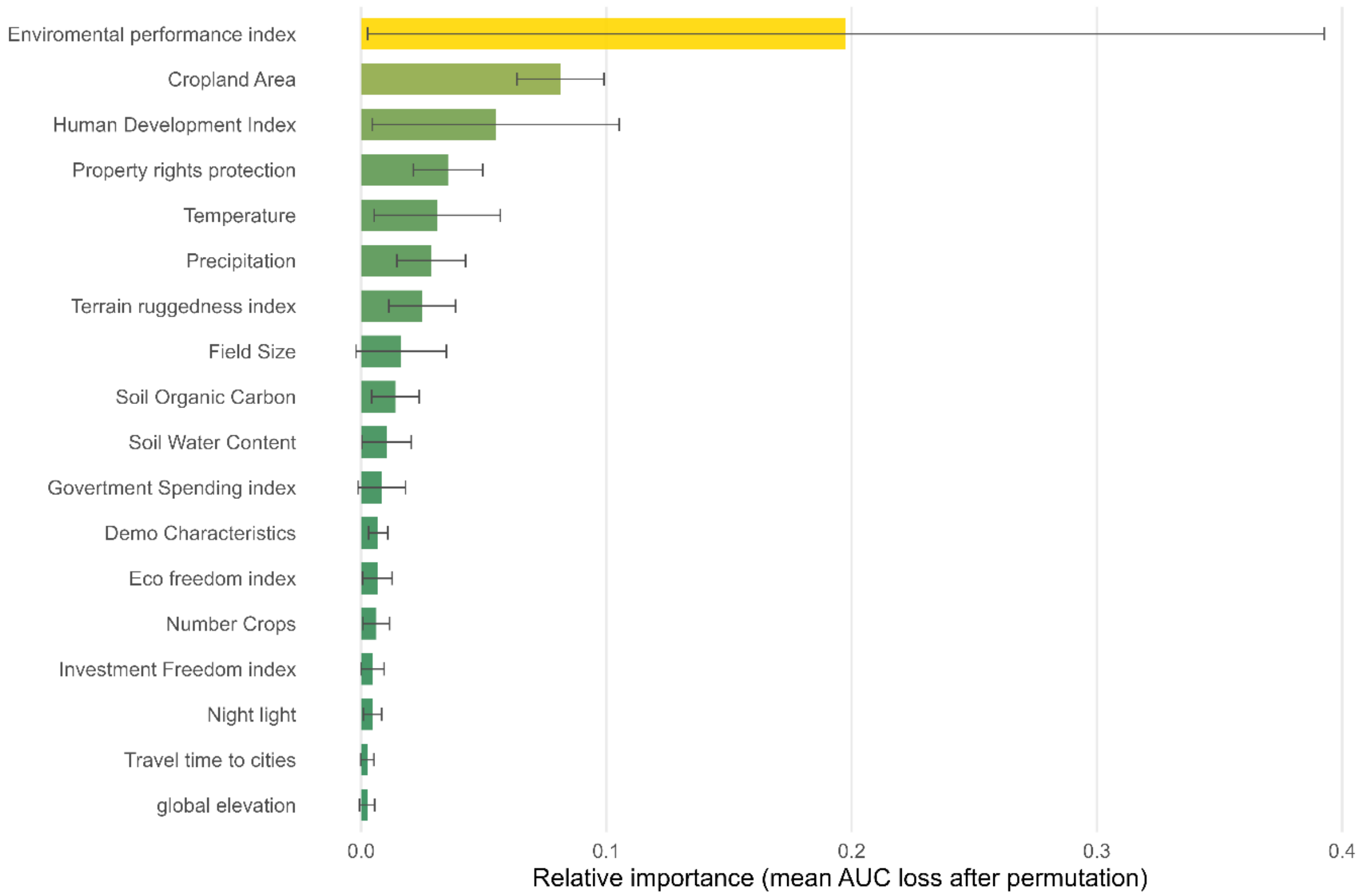

Fig. S2: Relative importance of predictor variables across the ensemble of geospatial machine learning models. Bars represent the ensemble-wide mean importance of each of the 18 predictor variables, estimated via a model-agnostic permutation procedure. Larger values indicate a greater contribution to predictive discrimination. Importance values are averaged across all 25 sub-models (5 algorithms × 5 spatial CV folds), providing a composite ranking that is robust to any single algorithm's rank. All analyses were performed using the sdm R package (1.2-56). (Per-algorithm importance with fold-level uncertainty (mean ± SD) is shown in Fig. S12; cross-model consistency of predictor rankings visualized as heatmap in Fig. S13.)

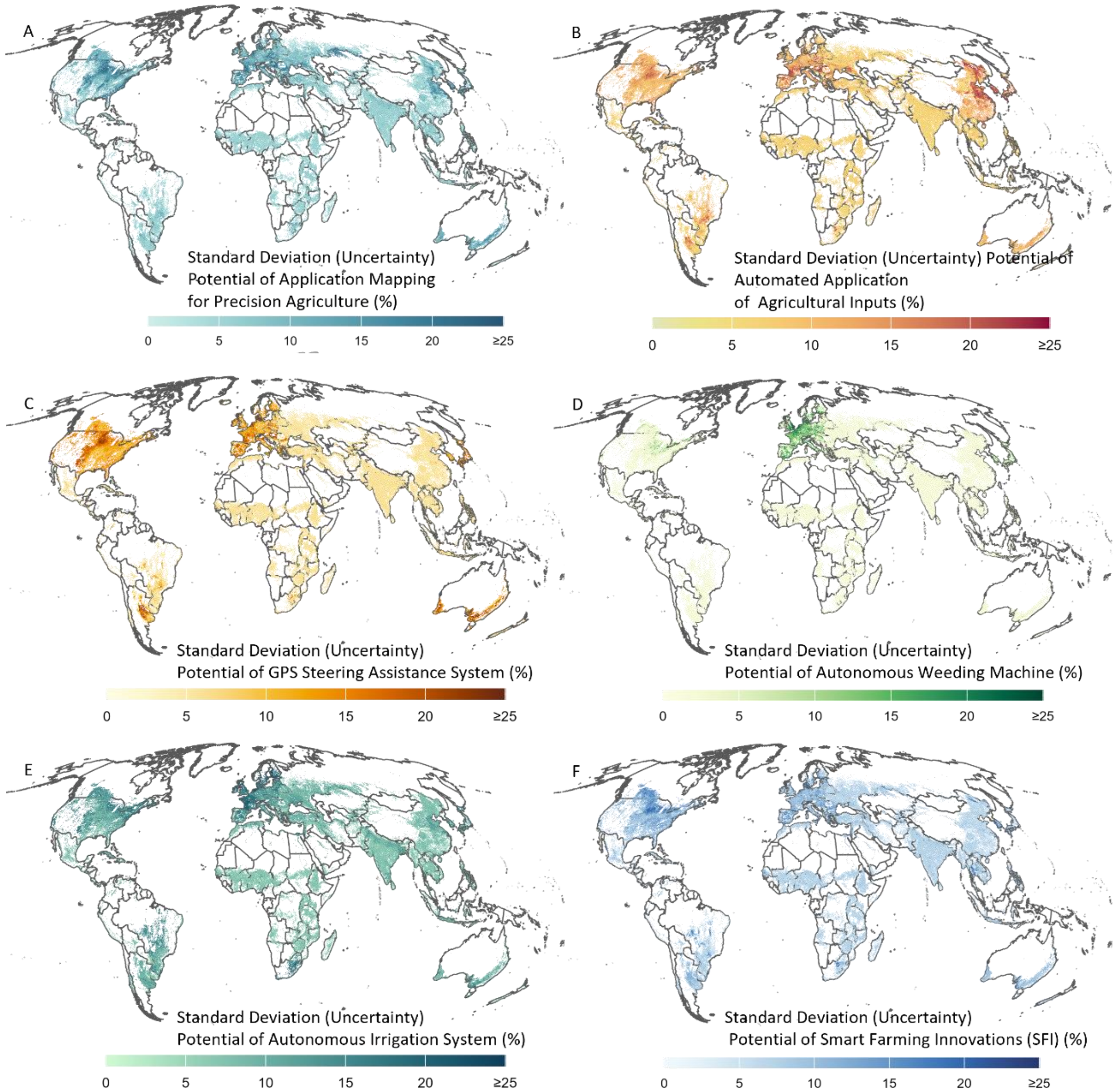

Fig. S3: Uncertainty maps (based on standard deviation across ML learners & Validations, n = 25). Panels A – E show specific innovations, while Panel F presents the overall spatial uncertainty for all innovations combined. The uncertainty is calculated at the pixel level as the standard deviation across 25 predicted layers derived from five models, which were developed using five-fold spatial block cross-validation

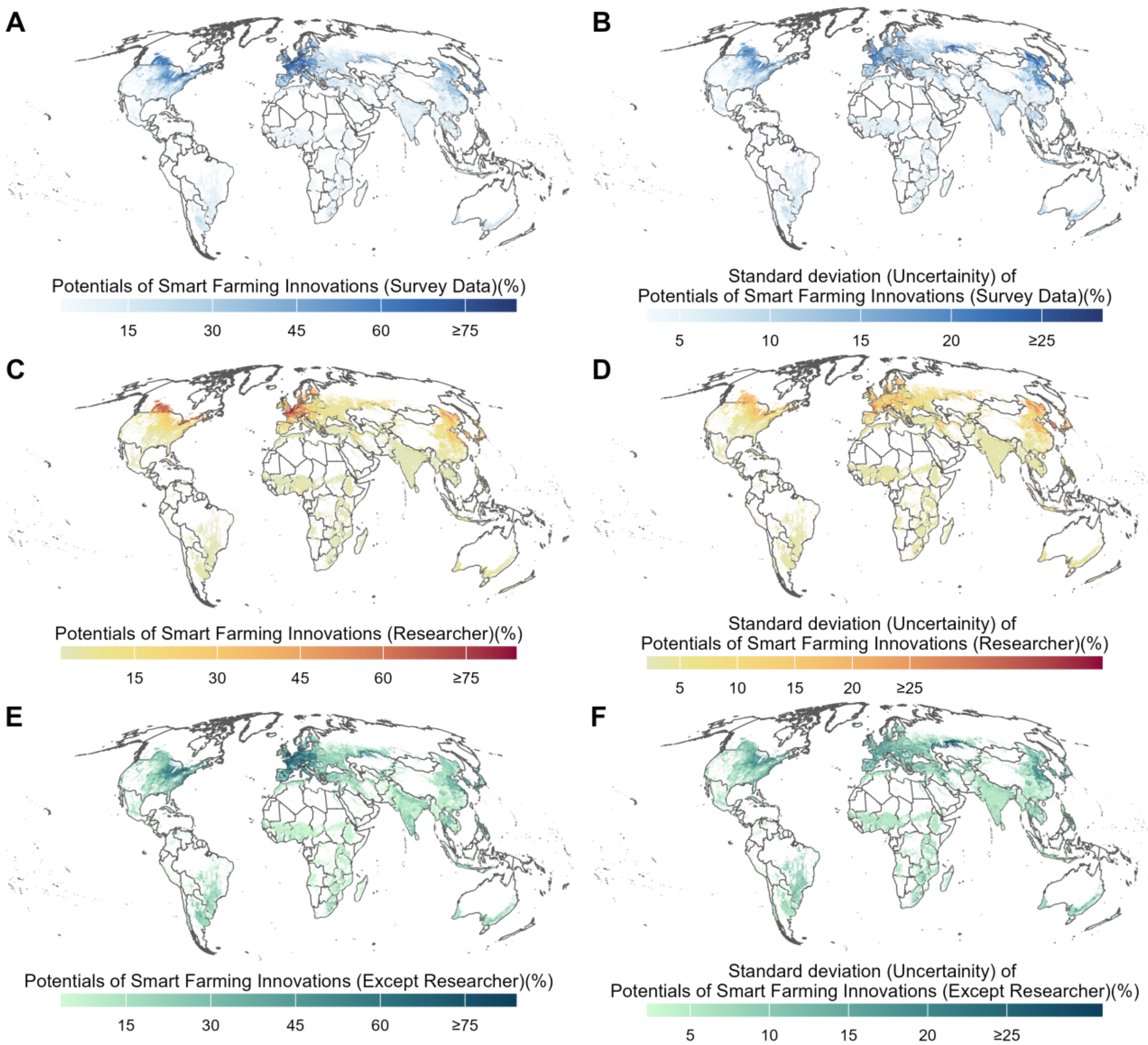

Fig. S4: Robustness Check of Data Sources. These maps are developed by using different sources of data. The map in panel A is developed by using all data (4422 data points), and the map in panel B is the standard deviation of the map in panel A across all ML models and validations. Panel C is the map developed by using only the data collected by the researcher (Research organization / University) from the survey (n = 2047, ~46%), and panel D is the standard deviation. Panel E is the map developed by using all data except researcher collected (n = 2375, ~54%) from farmers and agricultural technology companies sourced from the survey and panel F is the standard deviation of panel E

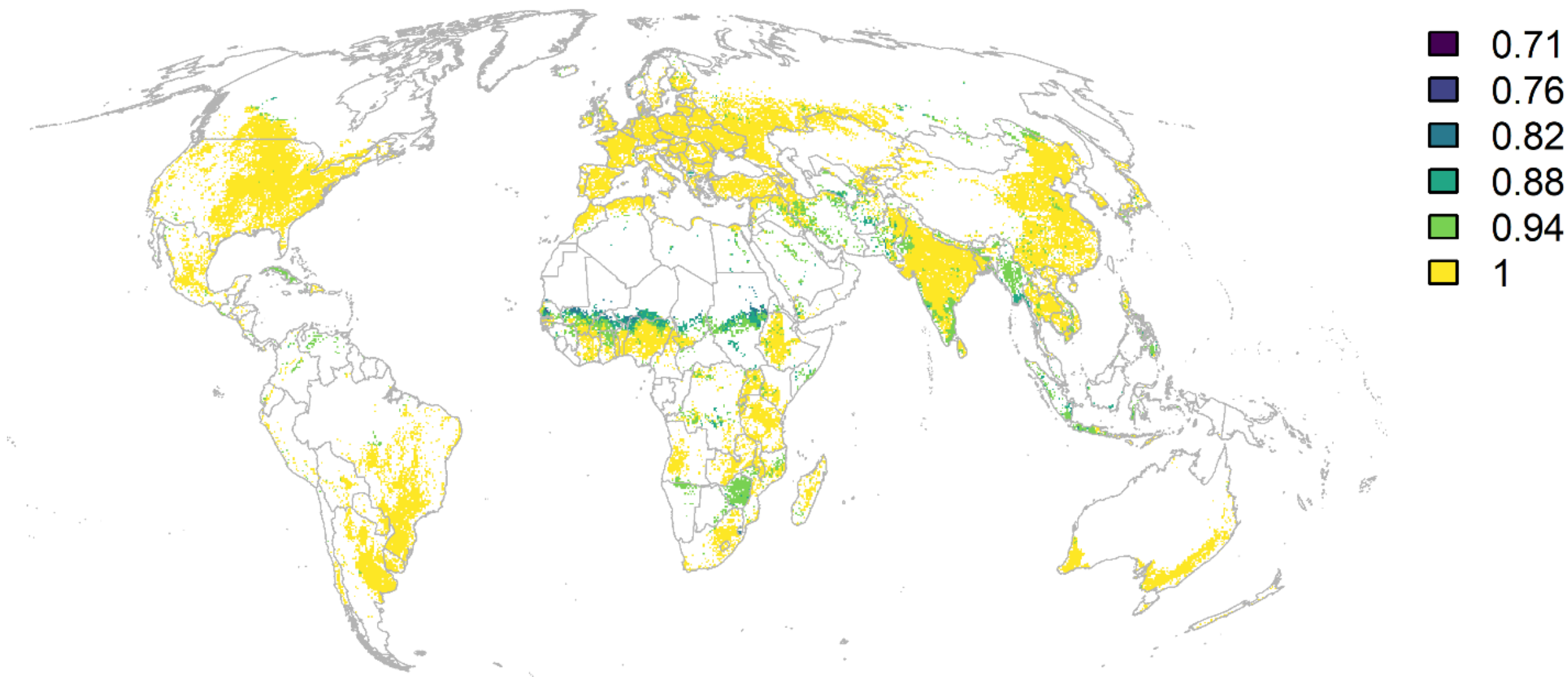

Fig. S5: Area of applicability map for Smart Farming Innovations. Grid cells are colored by their similarity index score (ranging from 0.71 to 1), with higher values indicating regions where model predictions are most reliable. Dark areas fall outside the area of applicability.

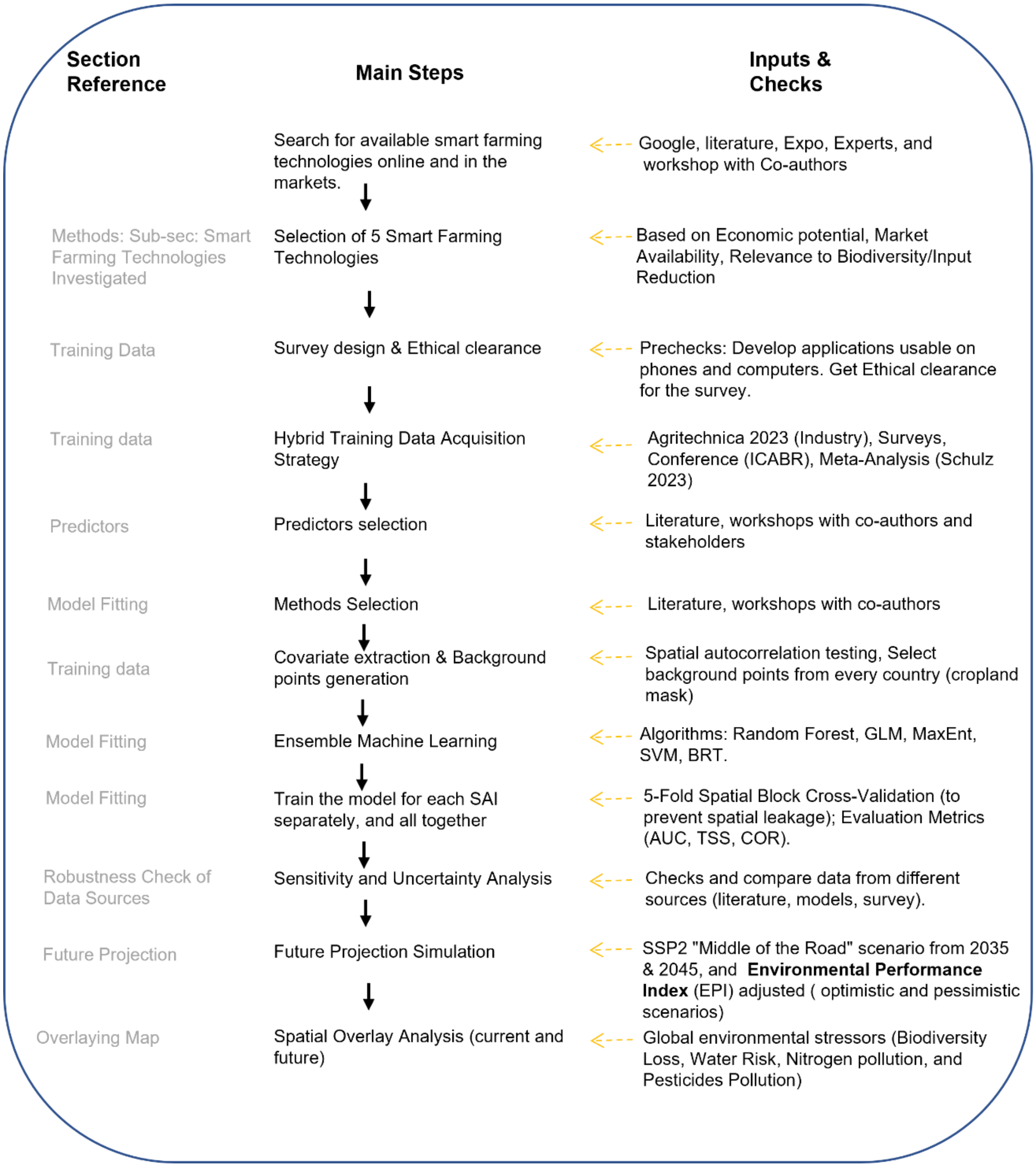

Fig. S6: Methodological workflow. The diagram outlines the sequential steps for survey design, predictor selection, ensemble modeling, and scenario-based spatial congruence analysis.

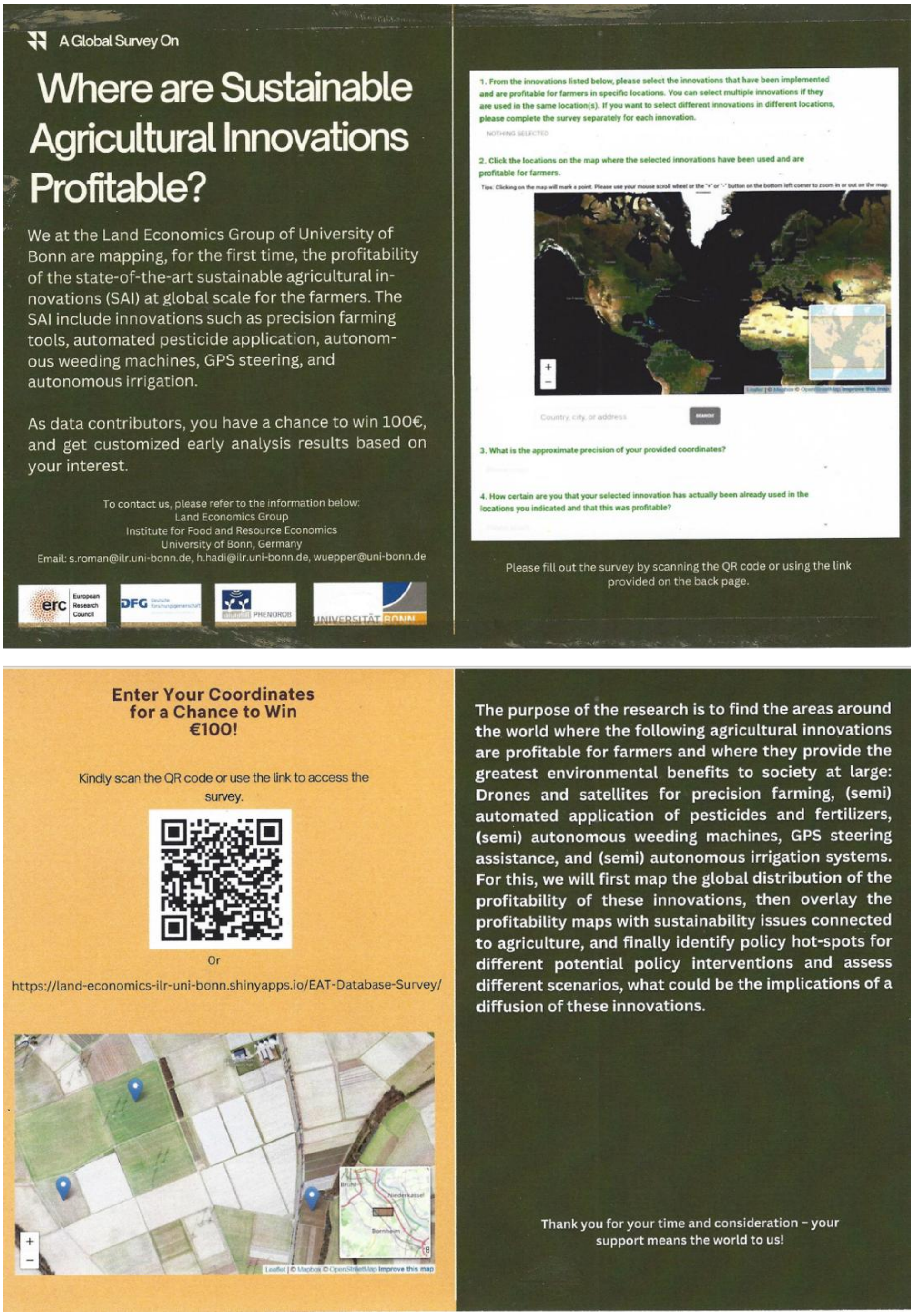

Fig. S7: Survey Leaflet. The leaflet was used to engage potential respondents at major agricultural technology events, including AGRITECHNICA 2023 (Hanover, Germany) and the ICABR 2024 conference. It was distributed directly to exhibitors and attendees showcasing relevant smart farming innovations to facilitate expert recruitment for the survey.

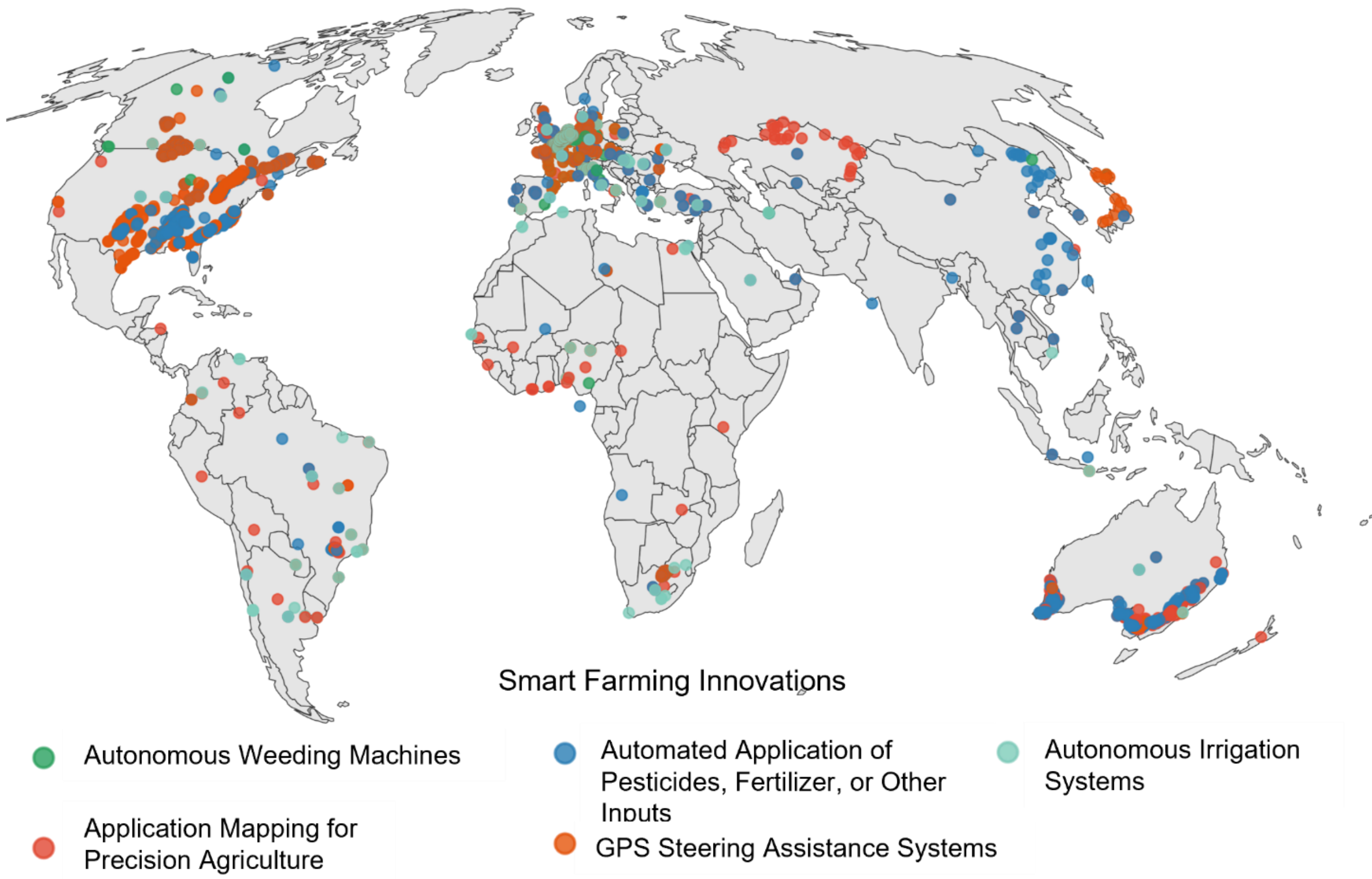

Fig. S8: The distribution of training points for all smart farming innovations.

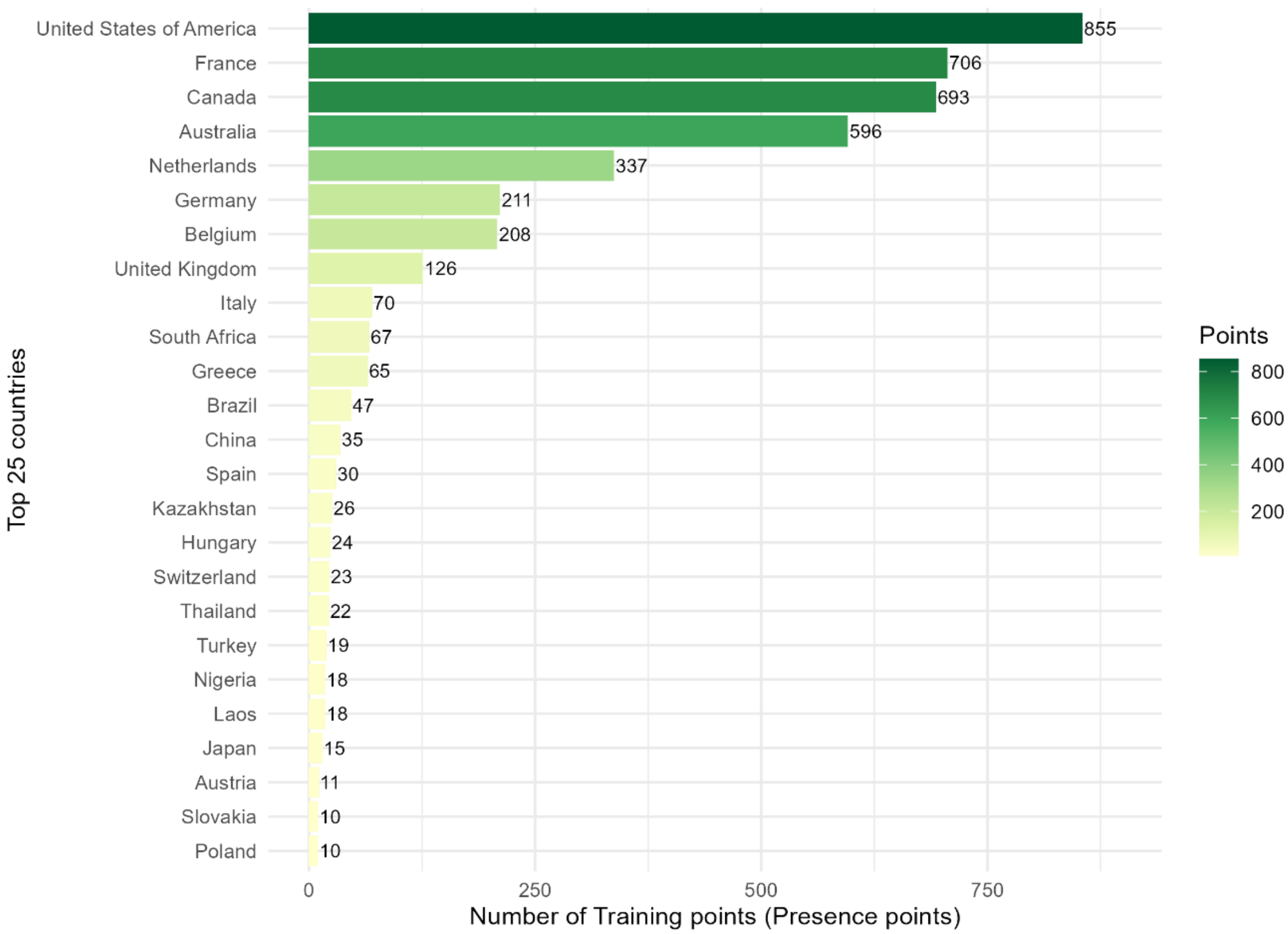

Fig. S9: Training points distribution for the top 25 countries out of 67 countries. Bars show the total number of presence points per country

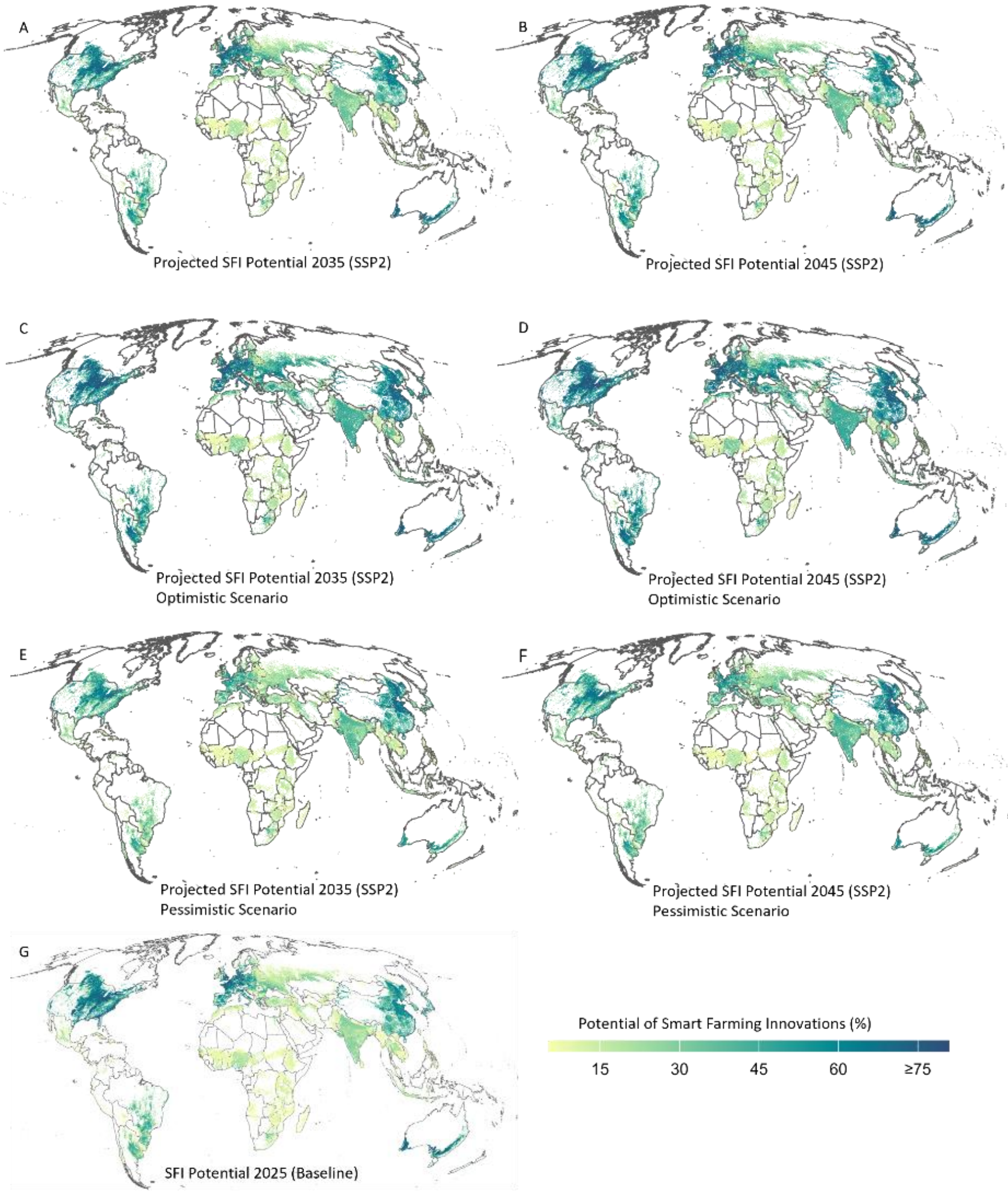

Fig. S10: **Global Economic Potential of Smart Farming Innovations (SFI) under the SSP2 "Middle of the Road" Pathway (2035 & 2045) with Varying Environmental Stringency**. A, B) Projections for 2035 and 2045, respectively, adjusting relevant variables according to SSP2 assumptions. C, D) High Environmental Stringency (Optimistic) Scenario for 2035 and 2045. In this scenario, the Environmental Performance Index (EPI) is adjusted to the maximum value currently (2024) observed within each country's World Bank income group. E, F) Low Environmental Stringency (Pessimistic) Scenario for 2035 and 2045. In this scenario, the EPI is adjusted to the minimum value currently observed in 2024 within each country's World Bank income group. Finally, G) is the baseline projection for 2025.

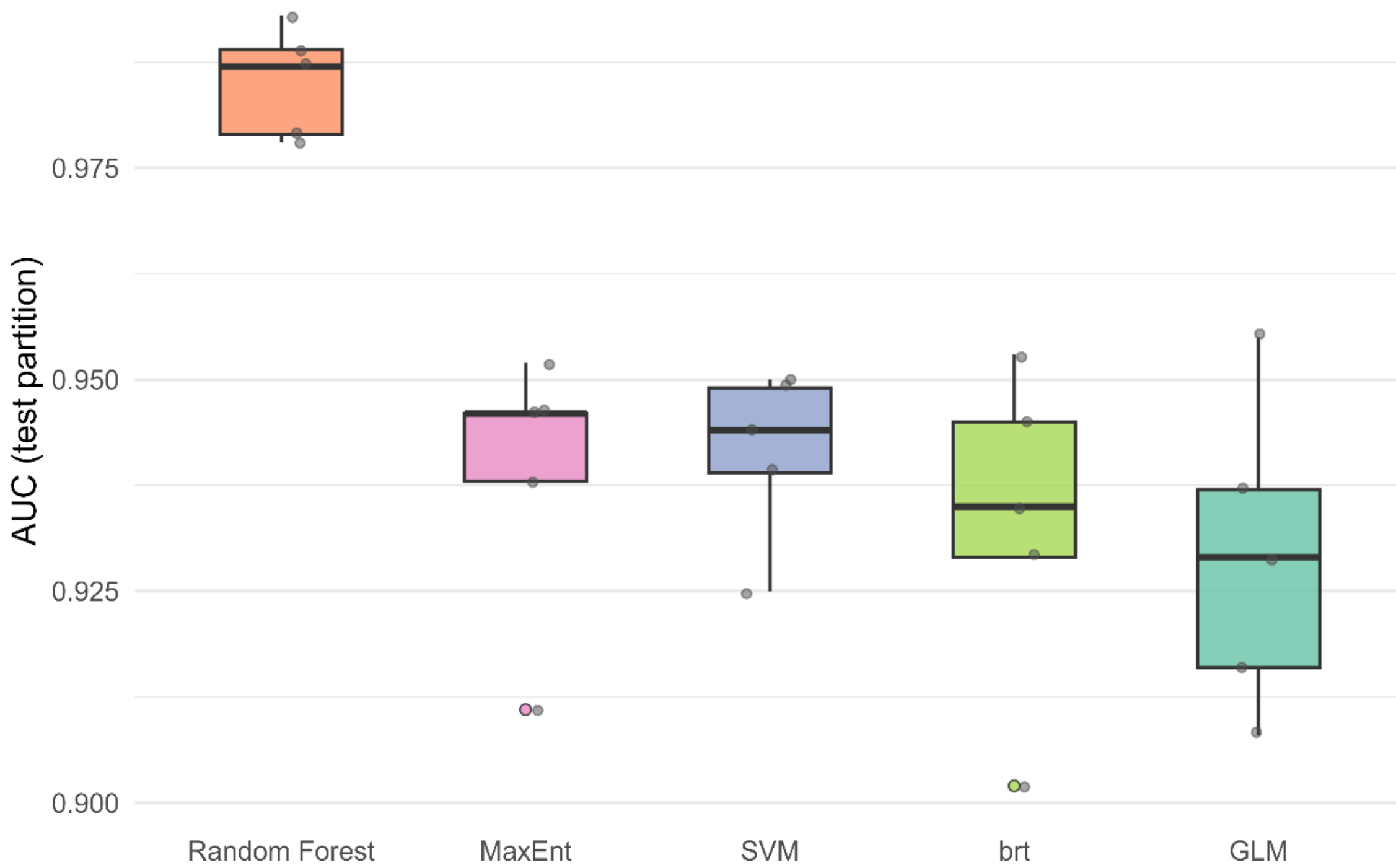

Fig. S11: AUC boxplot of smart farming innovations (SFI). Distribution of AUC scores across 5 spatial cross-validation folds per algorithm, evaluated on held-out test partitions. Boxes = inter-quartile range; horizontal line = median; dots = individual fold results

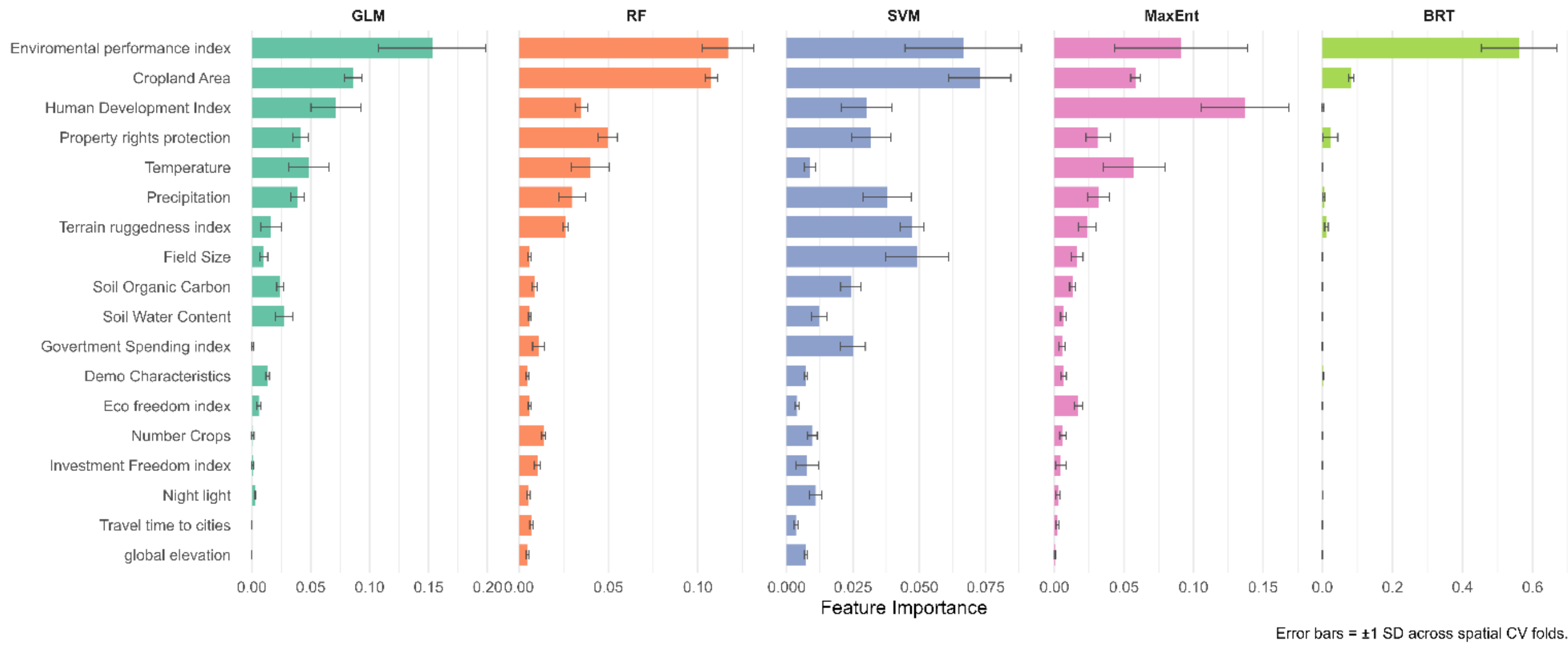

Fig. S12: Feature importance per algorithm (mean ± SD across 5 spatial CV folds), computed via permutation-based AUC loss within each algorithm. *Error bars = ±1 SD*

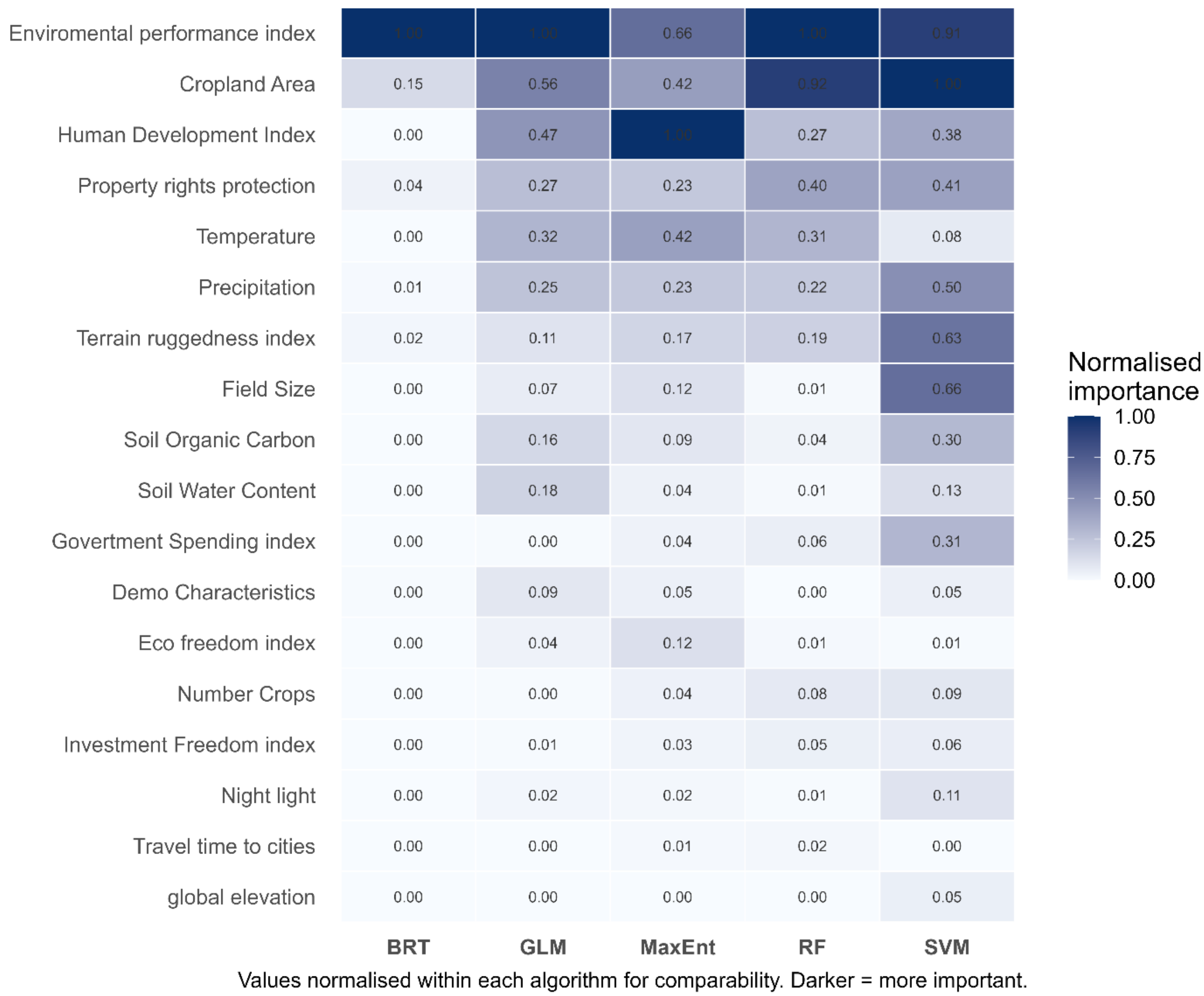

Fig. S13: Cross-model feature importance comparison (normalized 0–1 within each algorithm)*. Consistency in high-ranking variables across all five algorithms is consistent with the predictor set capturing an underlying process rather than algorithm-specific artifacts*

Tables

Table S1: Count of geolocations for each smart farming innovation

No:	Smart Farming Innovations	Count
**1**	Autonomous weeding machines	303
**2**	Autonomous irrigation systems	308
**3**	Application Mapping for Precision Agriculture	1201
**4**	GPS steering assistance systems	1271
**5**	Automated Application of Pesticides, Fertilizer, or Other Inputs	1339
**6**	Total	**4422**

Table S2: Overview of Features/Variables Influencing Smart Farming Technology Adoption and Diffusion

Variables	*Description*	*Unit*	*Year*	*Resolution*	*Source*
Economic freedom index	Measures countries' economic freedom by the security of property rights, inflation control, absence of excessive regulation, and various other indicators	Index (1 – 100)	2023	Country level	(1)
Human development index	A composite index assessing average achievements in education, income, and health	Index (0 – 1)	2015	~10 km grid	(2)
Soil organic carbon	The carbon stored in the soil's organic matter, contributing to soil health, fertility, and overall ecosystem functioning	Cg/kg	2021	~5 km grid	(3)
Soil water content	Indicates the volume of water retained in the soil, a critical factor for agricultural potential	Kilopascals (-10 kPa)	2021	~5 km grid	(3)
Precipitation of warmest quarter	The cumulative amount of rainfall or other forms of precipitation that an area receives on the warmest quarter over years	mm	1970-2000	~5 km grid	(4)
Temperature of the Warmest Quarter	The average temperature recorded in a given area over the warmest quarter over years	Degrees Celsius	1970-2000	~5 km grid	(4)
Terrain ruggedness index (TRI)	Calculated as the mean of the absolute differences in elevation between a focal cell and its eight neighboring cells	Index	2020	~250 m grid	(5)
Travel time to cities	The time required to reach the nearest densely populated area with at least 1,500 inhabitants as a proxy for market access	Minutes	2015	1 km grid	(6)
Demographics	Provides estimates of the working-age population (ages 15 – 64) and their densities (individuals per square kilometer), consistent with national census data	Number of persons (ages 15 – 64) per km^2	2010	~1 km grid	(7)
Property rights protection	Measures perceptions of the security of property rights, distinct from other aspects of the rule of law. This index synthesizes 18 individual indicators to offer a comprehensive view of property rights protection	Index (1 – 100)	2023	Country level	(8)
Number of crop types	Encompasses 42 individual crops and broader crop categories, offering a detailed represen-	Number of crop types per pixel (count)	2010	~10 km grid	(9)

	tation of global agricultural production and its distribution across various regions				
Cropland area	Refers to land used for the cultivation of annual and perennial herbaceous crops intended for human consumption, forage (including hay), and biofuel production	Percent of cropland per pixel	2019	3 km grid	(10)
Field size	Categorizes fields in very small (class 3506: <0.64 ha), small (3505: 0.64 – 2.56 ha), medium (3504: 2.56 – 16 ha), large (3503: 16 – 100 ha), and very large (3502: > 100 ha)	Categorical	2019	600 m grid	(11)
Elevation	Represents the average height of the land surface above sea level, derived from high-resolution digital elevation models.	m	2020	250 m (mean)	(5)
Environmental Performance Index	The Environmental Performance Index (EPI) offers a data-based overview of global sustainability efforts and outcomes.	Index	2023	Country level	(12)
Government Spending Index	Reflects the extent and efficiency of a country's government expenditures relative to its GDP, indicating how public spending may influence economic activity and the adoption of innovation.	Index	2023	Country level	(8)
Investment Freedom Index	The Investment Freedom Index measures the degree to which individuals and firms are free to invest capital both domestically and internationally without restrictions or government interference.	Index	2023	Country level	(1)
Nighttime light coverage	The Nighttime Light index uses satellite-based observations of artificial illumination at night to proxy human activity, economic development, and infrastructure presence across regions and countries.	Index	2018	30 arc-seconds (~1-km)	(13)

Table S3: Model Performance Metrics for modelling the Global Potential of Smart Farming Innovations. This table summarizes the evaluation of five geospatial machine learning modelling algorithms—Generalized Linear Model (GLM), Random Forests (RF), Support Vector Machines (SVM), Maximum Entropy (MaxEnt), and Boosted Regression Trees (BRT)—across five categories of smart farming innovations. For each technique and technology pairing, we report: Area Under the Receiver Operating Characteristic Curve (AUC), indicating the model's overall discriminative ability (0.5 = no better than random; 1.0 = perfect discrimination). Pearson's Correlation Coefficient (COR), measuring linear agreement between predicted suitability and observed profitable geolocations. True Skill Statistic (TSS), reflecting balanced accuracy by combining sensitivity and specificity (−1 to +1, where +1 is perfect). Deviance, quantifying model fit (lower values indicate better goodness-of-fit).

TECHNOLOGIES	MODEL (LEARNER)	AUC	COR	TSS	DEVIANCE
APPLICATION MAPPING FOR PRECISION AGRICULTURE	Generalized Linear Model (glm)	0.92	0.74	0.74	0.71
	Random Forest (rf)	0.97	0.82	0.84	0.67
	Support Vector Machine (svm)	0.93	0.76	0.77	0.68
	Maximum Entropy (MaxEnt)	0.92	0.73	0.72	0.78
	Boosted Regression Trees (brt)	0.92	0.74	0.76	0.85
AUTOMATED APPLICATION OF PESTICIDES, FERTILIZER, AND OTHER INPUTS	Generalized Linear Model (glm)	0.93	0.76	0.75	0.66
	Random Forest (rf)	0.98	0.87	0.90	0.54
	Support Vector Machine (svm)	0.94	0.77	0.78	0.67
	Maximum Entropy (MaxEnt)	0.94	0.77	0.77	0.72
	Boosted Regression Trees (brt)	0.93	0.77	0.77	0.83
AUTONOMOUS WEEDING MACHINES	Generalized Linear Model (glm)	0.97	0.80	0.89	0.30
	Random Forest (rf)	0.99	0.85	0.93	0.29
	Support Vector Machine (svm)	0.97	0.80	0.88	0.30
	Maximum Entropy (MaxEnt)	0.98	0.82	0.90	0.28
	Boosted Regression Trees (brt)	0.97	0.78	0.89	0.46
GPS STEERING ASSISTANCE SYSTEMS	Generalized Linear Model (glm)	0.95	0.80	0.80	0.59
	Random Forest (rf)	0.96	0.81	0.85	0.80
	Support Vector Machine (svm)	0.95	0.79	0.81	0.64
	Maximum Entropy (MaxEnt)	0.94	0.78	0.79	0.77
	Boosted Regression Trees (brt)	0.95	0.81	0.83	0.76
AUTONOMOUS IRRIGATION SYSTEMS	Generalized Linear Model (glm)	0.85	0.61	0.62	0.99
	Random Forest (rf)	0.95	0.76	0.81	0.87
	Support Vector Machine (svm)	0.86	0.64	0.71	0.89
	Maximum Entropy (MaxEnt)	0.81	0.51	0.55	1.32
	Boosted Regression Trees (brt)	0.87	0.65	0.66	1.02

Table S4: Smart farming innovation, descriptions, Primary environmental benefits, Current market availability/adoption and Modeled Potential & Future Expansion

Technology	Short description	Primary environmental benefits	Current market availability	Modeled Potential & Future Expansion
**Application mapping for precision agriculture**	Utilizes remote sensing (e.g., drones, satellites) to produce application guide maps tailored to specific field, soil, and crop conditions.	Creates a prescription to reduce over-application, nutrient runoff, and off-target pesticide use.	High commercial maturity; widely adopted in large-scale mechanized farming due to accessible satellite data and commercial drone services.	High baseline potential in North America and Western Europe; significant expansion projected in Eastern Europe and South America under SSP2
**automated input application**	Application machines (e.g., variable-rate tractors, robots, spraying drones) that precisely apply fertilizers/pesticides based on application maps.	Improves input efficiency and reduces pollution.	Commercially mature but capital-intensive; rapidly emerging adoption of variable-rate tractors, robots, and spraying drones	Strong overlap with current global hotspots of agrochemical pollution; highest adoption readiness in high-income regions, expanding into emerging economies.
**autonomous weeding machines**	AI-equipped and/or autonomous navigation robots designed to identify and mechanically/precisely remove weeds with minimal human intervention.	Strong potential to reduce herbicide use and protect non-target biodiversity.	Emerging to mature; highly dependent on advanced robotics, capital investment, and flat terrain, limiting immediate global ubiquity.	Strongest potential in regions with high labor costs and stringent environmental regulations (e.g., EU); future expansion closely tied to optimistic environmental governance scenarios.
**GPS steering assistance systems**	GPS-based auto-steer systems for agricultural machinery that allow for highly precise driving paths.	Reduces overlaps, fuel use, soil compaction; improves application accuracy.	Highly mature and arguably the most widely adopted smart farming technology globally; standard on most new large-scale tractors adopted in highly mechanized farming systems.	Widespread high potential across most mechanized agricultural regions globally; projected to expand rapidly into middle-income countries as economies grow.
**autonomous irrigation systems**	Precision irrigation systems utilizing sensors to adjust water application rates according to real-time soil and plant needs.	Reduces water use, runoff and associated nutrient loss; improves water-use efficiency.	Commercially available (e.g., smart pivots, drip systems); adoption heavily driven by regional water scarcity and high-value cropping.	High potential in both arid and temperate agricultural zones (e.g., Western and Eastern US); future expansion directly overlaps with regions facing severe and worsening water stress.

Table S5: Predictive performance (mean ± SD) across 5 spatially blocked CV folds, evaluated on held-out test partitions for Smart farming innovations (SFI).

Algorithm	N Folds	AUC (mean ± SD)	COR (mean ± SD)	TSS (mean ± SD)	Deviance (mean ± SD)
**Random Forest (RF)**	5	0.985 ± 0.006	0.877 ± 0.026	0.892 ± 0.029	0.510 ± 0.057
**SVM**	5	0.941 ± 0.010	0.793 ± 0.027	0.774 ± 0.028	0.638 ± 0.065
**MaxEnt**	5	0.939 ± 0.016	0.788 ± 0.038	0.764 ± 0.042	0.759 ± 0.064
**BRT**	5	0.933 ± 0.020	0.778 ± 0.035	0.759 ± 0.037	0.841 ± 0.040
**GLM**	5	0.929 ± 0.018	0.779 ± 0.040	0.758 ± 0.047	0.669 ± 0.088

Table S6. The Economics of Agricultural Technology Database (EATD) survey was administered as an online, self-completed instrument through an interactive Shiny application (Fig. S1). Respondents completed the items in the order shown below. The full wording and response options are reproduced here to document the instrument used for data collection

Order	Screen / item	Verbatim text shown to respondents	Response format	Response options, in displayed order
—	**Introductory screen**	**Instructions:** Please fill the survey in order from top to bottom. You can select multiple innovations if they are adopted in the same location(s). If you want to select different innovations in different locations, please complete the survey separately for each innovation. To save your answers, please click on submit your response when you are done. Please note that the web app has been optimized for viewing on a laptop computer.<br>**Privacy:** We guarantee that neither survey respondents nor individual farm can be identified from our published data.<br>**Lucky draw:** For a chance to enter the lucky draw to win 100 Euros in cash, please optionally leave your email address in the survey question.	Acknowledgement button	“Yes, I understood; proceed to survey”
1	**Innovation selection**	From the innovations listed below, please select the innovations that have been adopted and are profitable for farmers in specific locations. You can select multiple innovations if they are adopted in the same location(s). If you want to select different innovations in different locations, please complete the survey separately for each innovation.	Multiple selection	• Application Mapping for Precision Agriculture<br>• (Semi-) Automated Application of Pesticides, Fertilizer, or other Inputs<br>• (Semi-) Autonomous Weeding Machines<br>• GPS Steering Assistance Systems<br>• (Semi-) Autonomous Irrigation Systems
—	**Transition prompt**	Proceed to pin the locations and provide information about the above selected innovation(s) in this session?	Confirmation button	“OK”
2	**Location pinning**	Click the locations on the map where the selected innovations have been adopted and are profitable for farmers. Please pin the locations as precisely as you can.<br>**Map tip:** Clicking on the map will mark a point. Please use your mouse scroll wheel or the + or − button on the bottom left corner to zoom in or out on the map.<br>**Completion prompt:** Finish pinning the locations on the map where the selected innovations have been adopted and are profitable for farmers, and continue to the rest of the questions?	Interactive map; one or more points placed by the respondent. Coordinates recorded automatically.	Search field (placeholder “Country, city, or address”); “Search!” button; “Delete selected point” button; “Yes, finished” button.

Order	Screen / item	Verbatim text shown to respondents	Response format	Response options, in displayed order
3	**Coordinate precision**	What is the approximate precision of your provided coordinates?	Single selection	• Field<br>• Farm<br>• Village, City<br>• Region
4	**Certainty**	How certain are you that your selected innovation has actually been already adopted in the locations you indicated and that this was profitable?	Single selection	• Uncertain (<20%)<br>• Fairly certain (21%–40%)<br>• Moderately certain (41%–60%)<br>• Highly certain (61%–80%)<br>• Very certain (81%–100%)<br>• Completely certain (100%)
5	**Respondent details**	Your name<br>Your affiliation<br>Your email	Open text (optional)	—
—	**Submission**	—	Submit button	“Submit your response”

References

1. The Heritage Foundation, Index of Economic Freedom: All Country Scores | The Heritage Foundation. *Index of Economic Freedom | The Heritage Foundation* (2024). Available at: https://www.heritage.org/index [Accessed 19 August 2024].

2. M. Kummu, M. Taka, J. H. A. Guillaume, Gridded global datasets for Gross Domestic Product and Human Development Index over 1990-2015. *Sci Data* **5**, 180004 (2018).

3. L. Poggio, *et al.*, SoilGrids 2.0: producing soil information for the globe with quantified spatial uncertainty. *SOIL* **7**, 217–240 (2021).

4. S. Fick, R. Hijmans, WorldClim 2: New 1-km spatial resolution climate surfaces for global land areas. *International Journal of Climatology* **37** (2017).

5. G. Amatulli, D. McInerney, T. Sethi, P. Strobl, S. Domisch, Geomorpho90m, empirical evaluation and accuracy assessment of global high-resolution geomorphometric layers. *Sci Data* **7**, 162 (2020).

6. D. J. Weiss, *et al.*, A global map of travel time to cities to assess inequalities in accessibility in 2015. *Nature* **553**, 333–336 (2018).

7. Center for International Earth Science Information Network - CIESIN - Columbia University, *Gridded Population of the World, Version 4 (GPWv4): Basic Demographic Characteristics, Revision 11* (NASA Socioeconomic Data and Applications Center (SEDAC), 2024).

8. D. Wuepper, *et al.*, Agri-environmental policies from 1960 to 2022. *Nat Food* **5**, 323–331 (2024).

9. Q. Yu, *et al.*, A cultivated planet in 2010 – Part 2: The global gridded agricultural-production maps. *Earth System Science Data* **12**, 3545–3572 (2020).

10. P. Potapov, *et al.*, Global maps of cropland extent and change show accelerated cropland expansion in the twenty-first century. *Nat Food* **3**, 19–28 (2022).

11. M. Lesiv, *et al.*, Estimating the global distribution of field size using crowdsourcing. *Global Change Biology* **25**, 174–186 (2019).

12. Environmental Performance Index. *Environmental Performance Index*. Available at: https://epi.yale.edu/ [Accessed 30 April 2025].

13. X. Li, Y. Zhou, M. Zhao, X. Zhao, A harmonized global nighttime light dataset 1992–2018. *Scientific Data* **7**, 168 (2020).
